# AI-Driven Sensor Placement and Wireless Sensor Deployment: A PRISMA-Guided Systematic Review of Optimization Methods, Validation Maturity, and Benchmarking

**DOI:** 10.3390/s26216898

**Published:** 2026-10-30

**Authors:** Mohammad Sadman Tahsin, Haitham Y. Adarbah, Md Wahidur Rahman, Afzel Noore

**Affiliations:** AI-Based Autonomous System Research Lab, Department of Electrical Engineering and Computer Science, Frank H. Dotterweich College of Engineering, Texas A&M University-Kingsville, Kingsville, TX 78363, USA; md_wahidur.rahman@students.tamuk.edu (M.W.R.); afzel.noore@tamuk.edu (A.N.)

**Keywords:** wireless sensor network, sensor nodes, intelligent sensing, deployment, coverage, placement, artificial intelligence

## Abstract

Wireless sensor network (WSN) performance depends strongly on sensor deployment because node placement affects coverage, connectivity, energy consumption, localization, data reliability, and network lifetime. Although artificial intelligence (AI) is increasingly used for WSN deployment, the literature remains fragmented across methods, objectives, environments, metrics, and validation practices. This paper presents a PRISMA-guided systematic review and mapping of 51 primary studies on AI-driven WSN deployment, classified by AI technique, deployment objective, network environment, application domain, evaluation metric, validation method, methodological completeness, and reported limitations. Optimization-oriented AI dominates the field, particularly metaheuristics, swarm intelligence, and machine learning, while deep learning and reinforcement learning are emerging in predictive, adaptive, mobile, and application-specific settings. Swarm-intelligence methods are mainly used for coverage-centered static deployment, reinforcement learning for adaptive and mobile redeployment, and machine learning/deep learning methods for prediction-driven and application-specific placement. Coverage, connectivity, energy efficiency, and network lifetime remain the dominant objectives, whereas robustness, privacy, security, latency, edge feasibility, and lifecycle adaptation receive limited attention. Validation is a major methodological weakness, as most studies rely on simulation-only evaluation with limited use of real testbeds, field deployments, digital twins, long-term experiments, or standardized benchmarks. To address these gaps, the review introduces a validation-maturity perspective and benchmarking requirements emphasizing reproducible scenarios, explicit node assumptions, realistic sensing and communication models, common metrics, and shareable experimental artifacts. It also proposes an evidence-informed AI–Deployment–Validation Lifecycle Framework linking application requirements, environment modeling, objective formulation, AI-method selection, deployment optimization, validation, benchmarking, and lifecycle adaptation. The framework is intended as an organizing structure for future research rather than an empirically or expert-validated scoring instrument.

## 1. Introduction

Wireless sensor networks (WSNs) are widely used for distributed monitoring, automation, and decision support in cyber-physical environments. A WSN typically consists of spatially distributed sensor nodes that collect physical, environmental, or operational data and transmit them through wireless communication links to a sink, gateway, or processing unit. In such networks, sensor deployment is a critical design factor because node placement directly affects sensing coverage, network connectivity, energy consumption, communication reliability, deployment cost, and network lifetime [1]. Poor deployment can create uncovered regions, redundant sensing, disconnected nodes, excessive energy drain, packet loss, and unreliable data collection, even when the sensing hardware and communication protocols are technically adequate [2].

### 1.1. Background and Importance of WSN Deployment

WSN deployment is a fundamental network-design problem because node placement directly affects sensing coverage, connectivity, energy use, reliability, localization accuracy, maintenance cost, scalability, and network lifetime [3]. The spatial distribution of sensors determines whether the region of interest is adequately monitored, sensed data can reach the sink, and the network can operate effectively under environmental and operational constraints. Deployment should therefore be considered during early network design rather than as a secondary configuration step.

Its importance is particularly evident in practical applications. Smart agriculture requires placement that reflects soil, crop, irrigation, and microclimate variability, while healthcare and smart-home systems require reliable yet unobtrusive sensing. Industrial IoT, UAV-assisted sensing, disaster monitoring, battlefield surveillance, and emergency-response applications further require deployment strategies that account for interference, safety constraints, mobility, adaptability, and robustness.

### 1.2. Why AI Is Needed

Traditional grid-based, random, deterministic, and geometry-driven deployment methods are effective in relatively simple environments but become less suitable when obstacles, nonuniform sensing requirements, radio-frequency interference, terrain irregularities, node mobility, heterogeneous sensors, or strict energy constraints are present [4]. Such conditions are difficult to address using fixed placement rules or simplified mathematical assumptions.

WSN deployment is also inherently multi-objective. Improving coverage may increase energy consumption, reducing node count may weaken connectivity, and extending network lifetime may reduce sensing resolution. Effective deployment therefore requires trade-offs among coverage, connectivity, energy efficiency, lifetime, cost, reliability, localization accuracy, and application-specific sensing quality [5].

AI provides flexible tools for addressing these coupled and nonlinear challenges. Machine learning, reinforcement learning, swarm intelligence, evolutionary algorithms, Bayesian optimization, deep learning, and graph-based learning can explore large deployment spaces, learn from environmental feedback, adapt to changing conditions, and optimize placement under multiple constraints. These methods have increasingly been applied to coverage optimization, deployment planning, localization, routing, clustering, and energy-aware network design [6,7,8], making AI particularly relevant for uncertain, dynamic, and resource-constrained WSN environments.

### 1.3. Research Gap

AI-driven WSN deployment has received increasing attention, but the literature remains fragmented across AI methods, deployment objectives, assumptions, evaluation metrics, and validation practices. Studies employ techniques such as reinforcement learning, genetic algorithms, particle swarm optimization, fuzzy systems, Bayesian optimization, graph learning, deep learning, and hybrid metaheuristics, while targeting objectives including coverage, connectivity, energy efficiency, localization, cost, and network lifetime. This diversity makes it difficult to compare methods, assess evidence quality, and determine practical deployment readiness.

Existing reviews provide important foundations but do not fully address this fragmentation. Earlier reviews mainly examined WSN architecture, coverage, connectivity, and energy-efficient coverage protocols [9]. Broader AI and machine learning reviews considered deployment within wider WSN optimization problems rather than as the central focus [10]. More recent reviews on AI-based deployment, localization, metaheuristics, and deep learning offer closer coverage but still differ in scope, methodology, and analytical depth [11,12].

The main gap is therefore the lack of a review that jointly maps AI techniques, deployment objectives, application domains, validation methods, methodological-reporting completeness, benchmarking practices, and deployment readiness. Consequently, it remains unclear which AI methods dominate the field, which objectives and metrics are most commonly studied, how approaches are validated, and whether reported results are comparable across different network assumptions and experimental settings.

A systematic review is therefore needed to organize this evidence, assess methodological strengths and weaknesses, evaluate validation maturity, and identify future research priorities. Rather than aggregating heterogeneous results indiscriminately, such a review should connect AI methods with deployment objectives, performance metrics, application contexts, benchmarking practices, and real-world deployment readiness.

### 1.4. Contributions

This review makes the following contributions:

- It provides a systematic review of AI-driven WSN deployment studies, focusing on sensor placement, coverage enhancement, connectivity preservation, energy efficiency, localization-aware deployment, and application-specific deployment.
- It develops a structured taxonomy that links major AI techniques with deployment objectives and application contexts.
- It synthesizes the reviewed studies according to deployment objectives and performance metrics, including coverage, connectivity, energy consumption, network lifetime, deployment cost, redundancy reduction, localization accuracy, sensing reliability, packet delivery performance, computational cost, and recognition or reconstruction accuracy.
- It evaluates validation practices and methodological-reporting completeness across the literature by distinguishing simulation-based studies, numerical model-based validation, and real-world or application-specific deployments, with attention to benchmarking consistency and deployment readiness.
- It proposes a conceptual and evidence-informed AI–Deployment–Validation Lifecycle Framework that organizes application requirements, environment characterization, objective formulation, AI-method selection, deployment optimization, validation maturity, benchmarking, reproducibility, and lifecycle adaptation. The framework is presented as a synthesis-oriented research guide rather than as a validated scoring instrument.

## 2. Related Studies

This section positions the present review against prior surveys, reviews, and systematic mappings of WSN deployment and AI-based WSN optimization. The purpose is to identify how existing reviews frame the field and where a deployment-centered systematic synthesis remains needed.

### 2.1. Reviews on WSN Deployment

Early WSN surveys established deployment as a fundamental network-design issue because sensor limitations in energy, computation, communication, and storage directly affect coverage, connectivity, reliability, and network lifetime [1]. Later reviews examined deployment more specifically through coverage, connectivity, and energy efficiency. Cardei and Wu [13] focused on energy-efficient coverage, while Zhu et al. [4] emphasized the interaction between coverage and connectivity. More and Raisinghani [5] classified energy-efficient coverage protocols by sensing models, location information, neighbor discovery, and coverage control, and Elhabyan et al. [9] provided a broader taxonomy spanning deterministic, probabilistic, geometric, evolutionary, and swarm-based approaches.

More recent reviews extended deployment research to large-scale and smart-city settings. Abdulwahid et al. [10] systematically mapped deployment optimization algorithms for smart-city WSNs, particularly heuristic and metaheuristic approaches, while Du et al. [14] examined deployment and management issues in urban sensing systems. These studies provide important foundations but largely emphasize coverage, smart-city deployment, or classical optimization rather than the broader range of recent AI-driven methods, including reinforcement learning, deep learning, graph learning, Bayesian optimization, and hybrid approaches.

### 2.2. Reviews on AI-Based WSN Optimization

AI has also been widely reviewed in WSNs, although deployment is often treated as one task within broader network optimization. Alsheikh et al. [6] surveyed machine learning applications across routing, localization, clustering, data aggregation, event detection, and resource management, demonstrating the value of learning-based adaptation without making deployment the central focus.

Yan et al. [15] reviewed recent advances in energy-efficient routing protocols for wireless sensor networks. Their work addresses energy efficiency from the perspective of routing, whereas the present review focuses on AI-driven sensor placement, deployment, and redeployment. Although routing and deployment are related network-design problems, they involve different optimization decisions and methodological objectives.

More deployment-oriented reviews have since emerged. Osamy et al. [7] examined AI-based coverage, deployment, and localization, while Zaimen et al. [8] reviewed AI-based WSN deployment techniques and objectives such as coverage, connectivity, energy, cost, and lifetime. However, these reviews do not jointly examine AI technique, deployment objective, application domain, validation method, methodological-reporting completeness, benchmarking, and deployment readiness.

Related review streams focus on particular AI families. Srivastava and Mishra [16] and Houssein et al. [11] reviewed heuristic and metaheuristic methods for WSN problems, including deployment, localization, clustering, and energy optimization. These studies cover widely used methods such as genetic algorithms, particle swarm optimization, ant colony optimization, and related nature-inspired approaches, but provide less emphasis on reinforcement learning, deep learning, graph-based learning, Bayesian optimization, validation maturity, and edge feasibility.

Reinforcement learning and deep learning have also been reviewed in wireless-network contexts. Frikha et al. [17] surveyed reinforcement and deep reinforcement learning for wireless and mobile networking, while Qiu et al. [12] examined deep learning challenges in WSN deployment, including scalability, privacy, resource constraints, and energy efficiency. However, neither provides a systematic cross-family mapping of AI-driven WSN deployment evidence.

### 2.3. Positioning of the Present Review

Existing reviews provide important foundations in WSN architecture, coverage, connectivity, energy efficiency, machine learning, metaheuristics, reinforcement learning, and deep learning [1,4,5,6,7,8,11,16,17]. Their scopes, however, are generally broader than deployment or limited to specific algorithm families.

Related research also addresses specialized sensor-network environments and sensing modalities. Wang et al. [18] examined three-dimensional underwater wireless sensor networks, while Sheng et al. [19] considered wireless acoustic sensor networks. Betalo et al. [20] addressed UAV-assisted wireless sensor networks, and Zhu et al. [21,22] investigated wireless visual and directional sensor networks. These research directions highlight the importance of network geometry, sensing modality, mobility, and orientation constraints in application-specific sensor deployment. They provide complementary context for the present review, which focuses on the systematic classification and synthesis of AI-driven sensor placement and deployment methods across different WSN environments.

The present review treats AI-driven WSN deployment as the central unit of analysis. It examines sensor placement, redeployment, coverage, connectivity, localization-aware and energy-aware deployment, and application-specific positioning while jointly mapping AI technique, deployment objective, application domain, validation method, methodological-reporting completeness, benchmarking, reproducibility, edge feasibility, and deployment readiness. Table 1 summarizes the stated scope and analytical emphasis of selected prior reviews relative to the present review. The comparison is intended to clarify differences in review design and emphasis rather than to rank review quality or comprehensiveness.

The contribution of this review is primarily integrative. It combines AI-technique and deployment-objective mapping with methodological-reporting completeness, validation-maturity analysis, benchmarking and reproducibility considerations, and an assessment of deployment-readiness gaps. Edge feasibility is discussed as an emerging research and reporting consideration rather than as a systematically scored study-level dimension.

The review also proposes an AI–Deployment–Validation Lifecycle Framework linking application and environment characterization, objective formulation, AI-method selection, deployment optimization, validation, benchmarking, field transfer, and lifecycle adaptation. The framework is derived from recurring patterns and methodological gaps identified through the systematic mapping and narrative synthesis. It is conceptual and evidence-informed and has not been empirically tested, externally validated, or operationalized as a study-level scoring instrument.

## 3. Research Methodology

The Figure 1 summarizes the complete review procedure, beginning with protocol definition and research-question formulation, followed by Boolean search construction, database searching and snowballing, PRISMA-based identification, screening, and eligibility assessment [23]. It then shows the application of eligibility criteria, methodological-reporting completeness assessment, standardized data extraction, and final systematic mapping and narrative synthesis outputs, including AI-technique distribution, deployment-objective mapping, metric mapping, validation-method analysis, quality-level interpretation, research-gap identification, and benchmarking/validation-maturity recommendations.

### 3.1. Review Protocol

This review was conducted as a PRISMA 2020-guided systematic review with a systematic-mapping layer [24,25]. PRISMA 2020 was used to structure transparent reporting of the review rationale, search process, screening stages, eligibility decisions, and study flow, while PRISMA-S informed the reporting of search sources, search syntax, limits, and retrieval procedures [26]. Because the included studies differ substantially in network models, optimization objectives, validation settings, and outcome measures, the review was designed for structured mapping and narrative synthesis rather than quantitative meta-analysis.

The purpose of the review was to identify, classify, and critically synthesize studies that apply artificial intelligence to wireless sensor deployment problems, including sensor placement, redeployment, coverage optimization, connectivity preservation, and energy-aware deployment [27]. The review protocol was defined before the main screening stage and specified the research questions, search sources, search strings, eligibility criteria, study-selection workflow, quality-assessment rubric, data-extraction form, and synthesis strategy. This protocol-oriented approach improves reproducibility and reduces the risk of ad hoc selection or post hoc reframing of review decisions [28]. Table 2 summarizes the review protocol.

### 3.2. Research Questions

The review was guided by six research questions that connect directly to the findings, discussion, and critical-analysis sections. Table 3 summarizes the research questions of this review study.

RQ1 investigates the algorithmic landscape, including reinforcement learning, deep learning, machine learning, Bayesian optimization, swarm intelligence, evolutionary algorithms, graph-based learning, and hybrid metaheuristics. RQ2 examines whether studies focus on coverage, connectivity, energy efficiency, localization accuracy, network lifetime, cost, redundancy reduction, or sensing reliability. RQ3 identifies the deployment environments and application domains, such as smart homes, smart cities, industrial IoT, environmental monitoring, structural health monitoring, lunar exploration, and autonomous vehicle sensing. RQ4 examines the performance indicators used to evaluate deployment effectiveness. RQ5 assesses the methodological maturity of the literature by distinguishing simulation-based, dataset-based, numerical-model-based, and real-world validation. RQ6 synthesizes limitations and future directions.

These questions were intentionally framed to move beyond algorithm listing. Specifically, they were designed to reveal the dominant AI families, the optimization goals most frequently pursued, the maturity of validation practices, recurring methodological weaknesses, and the practical direction of the field. This structure is consistent with systematic-mapping guidance, which emphasizes classification, evidence structuring, and gap identification, while also aligning with systematic-review guidance that requires clearly specified review questions before study selection begins [29].

### 3.3. Search Strategy and Databases

The search strategy combined three concept blocks: network terms, deployment terms, and AI terms. The primary corpus covered 2015 to 2026. The lower bound was set to retain the earlier AI-oriented deployment studies present in the final evidence base while keeping the review focused on the modern period of intelligent and data-driven deployment research. Searches were limited to English-language publications and to peer-reviewed journal articles and full conference papers. The eligible document types were peer-reviewed journal articles and conference papers. Theses, editorials, book chapters, patents, posters, non-peer-reviewed reports, and inaccessible full texts were excluded. Google Scholar was not used as a primary database; instead, it was used only for backward and forward snowballing of highly relevant papers and review articles [30]. This combination of structured database searching and controlled snowballing is consistent with reproducible review practice. The detailed selection process is shown in Figure 2.

The search strategy was intentionally designed to capture studies specifically addressing AI-driven sensor placement, deployment, and redeployment, rather than the broader literature on AI applications in wireless sensor networks. To maintain this focus, the search combined three concept blocks covering WSN terminology, deployment-related terminology, and AI-based optimization techniques using the Boolean AND operator. This focused approach, together with the predefined publication period (2015–2026), document-type restrictions, and eligibility criteria, defined the boundaries of the evidence base. Consequently, the initial retrieval count of 124 records reflects the output of the specified search strategy rather than the overall volume of research on AI applications in WSNs.

The core Boolean search string used in this review was as follows:

**Search string:**

(“wireless sensor network” OR WSN OR “wireless sensor networks” OR “IoT sensor network” OR “sensor network”)

AND

(deployment OR placement OR “node placement” OR “sensor placement” OR

redeployment OR “coverage optimization” OR “target coverage”)

AND

(“artificial intelligence” OR “machine learning” OR “deep learning” OR

“reinforcement learning” OR “Q-learning” OR “genetic algorithm” OR “particle swarm optimization” OR “ant colony optimization” OR “bayesian optimization” OR metaheuristic OR “swarm intelligence” OR “graph learning” OR “fuzzy logic”)

To improve reproducibility, the same three concept blocks were preserved across all search platforms and translated into database-specific field syntax. Searches were run in title, abstract, and keyword fields where supported by the platform. On platforms with more limited field control, the equivalent advanced-search interface was used and relevance was then enforced during screening. PRISMA-S specifically emphasizes this level of search transparency and documentation.

A representative adaptation of the search syntax is shown below in Table 4.

The database searches were completed from May to July 2025, with the corresponding numbers of records used in this study are: IEEE Xplore = 22, ACM Digital Library = 4, ScienceDirect = 1, SpringerLink = 10, MDPI = 4, Nature = 5, Taylor & Francis = 1 and Wiley Online Library = 4. Table 4 documents the search expressions, field restrictions, and filters applied to the respective sources.

### 3.4. Article Selection Phase

The article-selection process followed the PRISMA sequence of identification, screening, eligibility assessment, and final inclusion. All retrieved records were exported in citation format, deduplicated, screened against predefined criteria, and then assessed at full-text level before final inclusion. PRISMA 2020 explicitly recommends transparent reporting of this full decision path and of reasons for exclusion at the eligibility stage.

All four authors participated in title and abstract screening and full-text eligibility assessment using the predefined criteria in Section 3.5. The screening was conducted collaboratively, with eligibility decisions evaluated jointly by the author team. Ambiguous cases were examined against the original full-text articles to support consistent application of the selection criteria. The process did not involve separate independent screening or formal inter-reviewer agreement estimation.

#### 3.4.1. Identification

The database and publisher-platform searches identified a total of 124 records before deduplication. This relatively focused retrieval count should be interpreted in the context of the predefined search boundaries, which required the combined presence of WSN-related, deployment-related, and AI-related terminology. The review specifically targeted studies in which AI techniques were applied to sensor placement or deployment problems, rather than general WSN optimization tasks. No records were identified through registers, and no records were removed using automated ineligibility tools.

Backward and forward snowballing were used as supplementary identification procedures. No additional unique records were identified through these procedures. Consequently, the 124 records shown in the PRISMA flow diagram represent database and publisher-platform retrievals before deduplication.

#### 3.4.2. Duplicate Removal

All retrieved records were exported into a reference-management workflow and checked for duplicate titles, author lists, publication years, DOIs, and venue names. Duplicate detection was first performed using reference-management software and then verified manually. A total of 29 duplicate records were removed before screening. An additional 14 records were removed at the prescreening stage for other reasons, including venue-related assessments and Scopus-indexing status. These considerations were not specified as eligibility criteria in Section 3.5 and are therefore distinguished from formal eligibility exclusions. Their potential influence on study selection is acknowledged as a limitation. No automated ineligibility tools were used.

#### 3.4.3. Title and Abstract Screening

After duplicate removal, 81 records were screened at the title and abstract level. At this stage, studies were excluded if they were not concerned with WSN or sensor-network deployment, did not use an AI-based method, or focused on other WSN tasks such as routing, clustering, localization, or data aggregation without a deployment component. Following title and abstract screening, 10 records were excluded and 71 reports were retained for full-text review.

#### 3.4.4. Full-Text Eligibility Assessment

A total of 71 full-text reports were sought for retrieval, of which 65 were successfully retrieved and assessed for eligibility. Fourteen reports were excluded: nine lacked sufficient methodological or evaluation information for reliable data extraction and appraisal, while five did not adequately meet the review scope. The nine reporting-based exclusions were based on the minimum extractability requirement specified in Section 3.5, rather than a threshold score from the seven-item reporting-completeness checklist or a formal assessment of methodological validity.

#### 3.4.5. Final Included Studies

The final analytical corpus consisted of 51 primary studies. These 51 papers formed the evidence base for the findings, discussion, and critical-analysis sections. Review papers, surveys, and systematic reviews identified during the background search were used only to support the related-work section and were not counted as primary studies in the final evidence corpus.

### 3.5. Selection Criteria

The selection criteria were defined before full-text screening to reduce selection bias and improve reproducibility. Inclusion criteria identified studies relevant to AI-driven WSN deployment, while exclusion criteria removed studies outside the review scope or lacking sufficient methodological detail.

**Inclusion Criteria**

- The study focuses on wireless sensor networks, IoT sensor networks, or sensor-node deployment contexts.
- The study applies an AI-based, machine learning, deep learning, reinforcement learning, evolutionary, swarm-intelligence, fuzzy, Bayesian-optimization, graph learning, or metaheuristic technique.
- The study addresses deployment, placement, redeployment, coverage optimization, target coverage, or sensor positioning.
- The study is an original peer-reviewed journal article or full conference paper.
- The study is written in English and the study falls within the selected publication period of 2015 to 2026.

**Exclusion Criteria**

- The study focuses only on routing, clustering, data aggregation, or cross-layer optimization without a deployment component.
- The study does not use an AI-based, machine learning, or metaheuristic method.
- The study is not related to WSN, IoT sensor-network deployment, or sensor placement.
- The study is a thesis, editorial, book chapter, patent, abstract-only paper, white paper, or non-peer-reviewed article.
- The full text is not available or the article is a duplicate, near-duplicate, or extended version of another included paper.
- The study does not provide sufficient methodological or experimental detail for reliable extraction.

### 3.6. Methodological-Reporting Completeness Assessment

A seven-item checklist was used to assess the completeness and transparency of methodological reporting in the included empirical studies (Table 5). The instrument was not designed as a formal risk-of-bias tool or as a comprehensive assessment of methodological validity. It evaluates whether a study clearly reports its deployment problem, AI method, objectives, evaluation metrics, comparator or baseline, evaluation setup, and limitations.

The checklist does not independently evaluate baseline-tuning fairness, statistical power, train-test leakage, hyperparameter-selection bias, robustness, external validity, code or data availability, or the realism of sensing and communication assumptions. These issues were considered qualitatively in the validation-maturity analysis and critical synthesis.

The checklist was applied only to empirical studies using simulation, datasets, numerical or physical models, controlled experiments, or field evaluation. Conceptual, review-oriented, and framework-based articles were not scored because criteria concerning experimental baselines and evaluation setup were not consistently applicable. These articles were retained only for contextual interpretation, methodological-gap identification, and future-direction analysis and were not used as evidence of algorithmic effectiveness or superiority.

Each item was scored as 1 only when the required information was explicitly and sufficiently reported; otherwise, it was scored as 0. Partial, implicit, or insufficiently detailed reporting was scored as 0. Total scores of 6–7 were classified as high reporting completeness, a score of 5 as medium reporting completeness, and scores of 0–4 as low reporting completeness. These categories indicate methodological-reporting completeness and should not be interpreted as formal risk-of-bias classifications.

For QA5, a score of 1 required at least one clearly identified comparator evaluated using compatible scenarios and performance metrics. For QA6, adequacy was interpreted according to the evaluation design. Simulation studies were required to report the main simulation environment and deployment parameters; dataset-based studies were required to identify the dataset and evaluation procedure; numerical- or model-based studies were required to report the main model assumptions and parameters; and physical studies were required to describe the hardware, environment, and experimental procedure. For QA7, a score of 1 required at least one concrete methodological or applicability limitation rather than only a generic statement about future work.

#### Methodological-Reporting Completeness Assessment Results

To make the reporting-completeness appraisal transparent, each of the included studies was scored against the seven quality-assessment questions in Table 6. A score of 1 was assigned only when the item was explicitly and sufficiently reported in the paper. A score of 0 was assigned when the item was absent, unclear, only indirectly discussed, or insufficient for replication. Borderline cases were handled conservatively: if the evidence was partial but not enough to support reliable extraction or comparison, the item was scored as 0. This strict binary scoring was used to avoid overstating the methodological strength of studies with limited reporting detail.

Table 6 summarizes the methodological-reporting completeness of the included studies. These scores indicate the availability and transparency of reported information rather than methodological validity, risk of bias, or algorithmic effectiveness. A high score does not establish reliable performance, while a low score does not necessarily indicate an ineffective method. Accordingly, the scores were treated as descriptive indicators of reporting completeness rather than measures of evidence strength or deployment readiness.

The nine reports excluded during full-text eligibility assessment lacked sufficient information for reliable extraction and appraisal. Their exclusion was not based on the seven-item reporting-completeness score. In contrast, included studies with low reporting-completeness scores met the minimum information requirements and were retained for descriptive or contextual synthesis. Thus, reporting completeness was used to qualify the strength of the included evidence rather than to determine eligibility through a numerical quality threshold.

All four authors participated in study classification and methodological-reporting completeness assessment using the predefined extraction dimensions and seven-item checklist. The assessment criteria were applied consistently across the included studies. Borderline scoring decisions were re-examined against the original full-text articles and the item-specific checklist definitions during manuscript revision. Individual scoring decisions were compared, and discrepancies were resolved through discussion and consensus before finalizing the study-level scores.

Interpretation of algorithmic effectiveness was based on the findings reported within individual studies, considering experimental assumptions, baseline comparisons, evaluation metrics, and validation conditions rather than reporting-completeness scores. Claims of deployment readiness were considered separately in relation to validation realism, physical testing, and operational evidence. Consequently, reported performance improvements were interpreted within their study-specific settings and were not treated as proof of generalizable superiority or real-world readiness. Conceptual and framework-oriented studies were retained for contextual interpretation and research-gap identification rather than direct performance comparisons.

### 3.7. Data Synthesis

A formal meta-analysis was not performed because the included studies do not report a common effect measure and differ substantially in network assumptions, sensing models, communication constraints, optimization objectives, simulation tools, benchmark baselines, and outcome variables [82]. Under such conditions, a pooled quantitative effect estimate would be methodologically weak and potentially misleading. Instead, the review used descriptive statistics, systematic mapping, cross-method comparative synthesis, and thematic narrative analysis.

The synthesis proceeded in four layers. First, descriptive mapping was used to summarize publication trends, dominant AI techniques, deployment objectives, application domains, evaluation metrics, and validation methods. Second, cross-classification was used to connect AI technique families with deployment objectives, performance metrics, application domains, and validation practices. Third, a cross-method comparative evidence synthesis was conducted to compare the methodological and performance-evaluation patterns of major AI families, including swarm intelligence, reinforcement learning, machine learning, deep learning, Bayesian optimization, graph-based methods, and hybrid approaches. This comparison focused on evidence frequency, dominant objectives, common metrics, validation maturity, baseline use, and reported direction of improvement within individual studies rather than pooled percentage improvements across incompatible studies. Fourth, thematic analysis was used to synthesize methodological strengths, recurrent weaknesses, research gaps, and future directions.

For AI-technique classification, a hierarchical, multi-label approach was adopted to distinguish algorithm families from supporting techniques and hybrid implementations. Genetic algorithms were grouped within evolutionary optimization, while swarm-based and other nature-inspired metaheuristics were distinguished as a separate optimization group. Machine learning, deep learning, and reinforcement learning were identified according to their reported methodological components. Fuzzy logic and graph-based techniques were treated as cross-cutting components, while hybrid methods were identified as an additional descriptor when multiple techniques were combined. Accordingly, a study could be assigned multiple technique tags, and category frequencies were not intended to be mutually exclusive or additive.

Deployment objectives were similarly mapped using non-mutually exclusive categories. Each study was counted once within every objective category it addressed, allowing multiple assignments for multi-objective deployment methods. A primary objective refers to the principal deployment goal, whereas secondary objectives represent additional goals, constraints, or performance considerations. However, the present mapping did not systematically assign these priority levels to individual studies. Consequently, the reported frequencies indicate how often objectives were addressed, not how often they served as primary optimization targets.

This layered synthesis is appropriate for an interdisciplinary engineering corpus in which the most meaningful output is not a single pooled effect size, but a structured understanding of where the field is concentrated, how different AI families are used, which validation practices dominate, where evidence remains weak, and which methods appear most suitable for specific deployment settings.

## 4. Findings and Discussion

### 4.1. Observations

The descriptive synthesis below is based on manual coding of the 51 included studies reported in the review protocol. For the technique, objective, environment, and validation tables, several categories are non-mutually exclusive because a number of studies combine multiple AI methods or optimize more than one deployment objective.

#### 4.1.1. Publication Trend

The publication trend shows that AI-driven wireless sensor deployment has gained stronger attention in recent years. This suggests that AI-driven WSN deployment is currently an active and expanding research direction. The increase after 2020 is also consistent with the broader shift from classical deployment heuristics toward adaptive, learning-based, and application-specific deployment strategies.

#### 4.1.2. Distribution by AI Techniques

Table 7 presents the distribution of AI techniques using a hierarchical, multi-label classification. The taxonomy distinguishes optimization and learning-based method families from supporting techniques, hybrid implementations, and other contextual approaches. Genetic algorithms are grouped with evolutionary algorithms, while swarm-based and nature-inspired metaheuristics form a separate optimization group. Fuzzy logic and graph-based techniques are treated as cross-cutting components rather than independent, mutually exclusive algorithm families. Hybrid methods are identified separately to indicate the combination of multiple techniques. For example, fuzzy logic is combined with PSO in [48], while DRL-GNN integrates reinforcement learning and graph learning. Therefore, individual studies may appear in multiple categories, and the reported frequencies represent technique occurrences rather than exclusive study groups.

The expanded corpus shows that optimization-oriented AI remains the dominant direction in AI-driven WSN deployment research. Swarm intelligence and nature-inspired metaheuristics are especially common, including PSO-based methods [39,44,48], artificial bee colony optimization [63], whale optimization [66], crested porcupine optimization [55], glowworm swarm optimization [67], jellyfish optimization [69], and other biologically inspired optimization strategies [57,67]. These methods are mainly used for coverage maximization, connectivity preservation, energy-aware deployment, and network-lifetime improvement.

Machine learning and deep learning methods are also increasingly visible, particularly in predictive placement, smart-home deployment, structural monitoring, activity recognition, energy forecasting, localization-aware deployment, topology identification, smart-city planning, and application-specific sensor placement [35,40,46,47,51,52,54,66,70,73,79,80,81]. Compared with classical metaheuristics, these methods are more often used when historical data, environmental observations, motion data, structural-response data, or topology information are available.

Reinforcement learning and deep reinforcement learning constitute a smaller but important methodological group in AI-driven WSN deployment. These approaches are particularly relevant to adaptive and sequential placement problems, including mobile deployment, dynamic coverage control, and topology-aware redeployment. As summarized in Table 7, the category contains 10 distinct studies. Their practical effectiveness depends on reward formulation, state and action representation, computational requirements, and the realism of training and validation environments.

#### 4.1.3. Distribution by Deployment Objective

Table 8 summarizes the frequency of deployment objectives addressed across the 51 included studies. Because many studies consider multiple objectives, the categories are non-mutually exclusive. Coverage maximization appears most frequently (31 studies), followed by energy efficiency, network lifetime, connectivity, and other deployment objectives. However, these counts do not distinguish between primary and secondary objectives. For example, coverage may be the principal optimization target in one study but an additional objective or performance consideration in another. Therefore, the reported frequencies reflect objective prevalence rather than priority or relative importance.

Coverage is the most frequently represented objective in the reviewed studies because monitoring the intended sensing region or target set is a fundamental WSN deployment requirement. However, the distribution also shows that deployment research increasingly treats coverage as only one part of a broader optimization problem. Many studies jointly optimize coverage with connectivity, energy consumption, network lifetime, cost, latency, robustness, redundancy, or task-specific sensing quality [31,32,34,48,49,56,58,61,64,65,67,69]. This confirms that AI-driven deployment should be interpreted as a multi-objective optimization problem rather than a single-objective area-coverage problem.

The expanded objective distribution also shows a clear separation between general WSN deployment objectives and application-specific sensor-placement objectives. General WSN studies emphasize coverage, connectivity, energy efficiency, and lifetime, while application-specific studies focus on objectives such as flood detection, human activity recognition, plantar-pressure trajectory estimation, posture reconstruction, pollutant detection, structural damage identification, spatiotemporal modeling accuracy, pipeline damage detectability, and topology observability [59,60,62]. These application-specific objectives define deployment effectiveness for their respective sensing tasks, although their relative priority cannot be inferred from the frequency-based classification.

#### 4.1.4. Distribution by Application Domain

The selected studies cover a broad range of deployment environments and application domains, from conventional 2D and static WSN models to 3D deployment, mobile and adaptive networks, underwater monitoring, industrial IoT, smart-city infrastructure, healthcare-oriented sensing, wearable systems, structural health monitoring, power systems, and safety-critical infrastructure monitoring. Because several studies address more than one deployment context, the categories in Table 9 are not mutually exclusive. For example, a study may be counted under both 2D WSN and smart-city deployment, or under both underwater monitoring and mobile deployment.

The application distribution also shows that sensor deployment research is moving beyond generic area-coverage problems. Healthcare, assisted-living, wearable sensing, and human activity recognition studies emphasize sensor placement for recognition accuracy, motion reconstruction, comfort, and reduced sensor count rather than only spatial coverage [40,47,53,54,59,62,63]. Structural health monitoring, modal testing, pipeline monitoring, and distributed process modeling emphasize information quality, damage detectability, reconstruction accuracy, and model observability [46,51,70,71,72]. Water infrastructure and power-system studies further broaden the deployment problem toward flood detection, and topology observability [52,60,74].

Overall, the reviewed studies remain concentrated around simplified 2D and static WSN settings, but the application base is becoming more diverse. This expansion is important because deployment objectives differ across domains. A method designed for area coverage in a planar WSN may not directly transfer to underwater networks, wearable sensing, structural monitoring, smart production, or power-system observability. This reinforces the need to evaluate AI-driven deployment methods not only by algorithmic performance, but also by application context, environmental realism, validation method, and deployment constraints.

#### 4.1.5. Distribution by Validation Method

Validation practice is one of the clearest indicators of methodological maturity, as shown in Table 10. Because several studies use more than one validation strategy, the categories in the table are not mutually exclusive. For example, a study may combine simulation with benchmark-based evaluation, numerical modeling, or real-system validation. These categories describe distinct and potentially overlapping validation attributes rather than mutually exclusive stages of a single ordered maturity scale. Evaluation medium, data source, physical realism, benchmarking, and analytical verification are therefore treated separately.

The selected studies show a strong dependence on simulation and custom computational evaluation. Simulation is useful for early-stage algorithm testing because it allows controlled comparison across deployment strategies, node densities, objective functions, and network constraints. However, simulation-only validation provides limited evidence of practical transferability. Methods evaluated only under simplified assumptions may not perform the same way under real environmental uncertainty, hardware constraints, radio irregularity, sensor failure, communication interference, calibration error, maintenance limits, or long-term energy depletion.

Benchmark-based, dataset-based, and numerical-model-based evaluations provide different forms of evidence by connecting deployment decisions to reference datasets, domain models, or task-specific performance measures. Their evidentiary value depends on the realism, independence, calibration, and relevance of the underlying benchmark, dataset, or model, and they should not automatically be considered stronger than simulation-based evaluation.

Validation categories are non-mutually exclusive and should not be interpreted as ordinal maturity levels. Of the 33 studies listed under simulation or custom computational evaluation, 26 are listed exclusively in that category, while 7 also appear in at least one other validation category. The field-deployment study [47] is also included among the five studies coded as real testbed or controlled physical experiments; therefore, these categories represent five distinct physical/field studies rather than six. Counts describe the validation modes identified during extraction and should not be interpreted as mutually exclusive study counts.

For interpretation, validation evidence can be characterized along several separable dimensions: whether data are synthetic or measured; whether physical models are generic or application-calibrated; whether evaluation is software-based or involves physical hardware; whether the optimized sensor layout is physically deployed; whether testing occurs in a laboratory/controlled or operational setting; whether validation uses an independent environment, dataset, or subject population; and the duration of observation. These dimensions are conceptually distinct and are not combined here into a numerical maturity score. Because these attributes were not uniformly reported or extracted across all included studies, the present review does not retrospectively assign study-level scores for them.

#### 4.1.6. Cross-Method Comparative Evidence Synthesis

The reviewed studies were compared across major AI method families to identify their dominant deployment uses, evaluation metrics, validation patterns, and application contexts. Because the studies differ substantially in deployment areas, node densities, sensing and communication models, energy assumptions, simulators, datasets, baselines, and application constraints, the analysis does not rank methods using pooled performance percentages. Instead, it provides a structured cross-method comparison of where each AI family is most commonly applied and how strongly it is validated.

The comparison follows the hierarchical, multi-label classification introduced in Section 4.1.2. The method families represent analytical groupings rather than mutually exclusive categories. Supporting techniques, such as fuzzy logic, and composite approaches, such as hybrid AI methods, are discussed separately to clarify their methodological roles. The studies listed in the comparative synthesis are illustrative examples rather than exhaustive category memberships.

Although implementation details vary across the reviewed studies, the major AI approaches follow recognizable solution principles. Evolutionary and swarm-based methods explore candidate sensor configurations and optimize placement according to predefined objectives and constraints. Machine learning and deep learning approaches use learned representations or predictions to support data-driven placement decisions. Reinforcement learning formulates deployment or redeployment as sequential decision-making, where actions are selected based on network states and reward feedback. Hybrid approaches integrate complementary techniques to address complex or multi-objective deployment requirements. The resulting deployment decisions are evaluated using application-relevant metrics, such as coverage, connectivity, energy efficiency, or sensing accuracy. These general methodological principles complement the comparative analysis in Table 11 and the conceptual lifecycle stages; they do not imply an identical implementation pipeline across all studies.

The representative studies in Table 11 have been aligned with the technique assignments in Table 7. In particular, deep-learning-based optimization is distinguished from reinforcement learning, while swarm-based optimization and general machine learning approaches are not automatically classified as deep learning. Multiple labels are retained only when the corresponding methodological components are supported by the study description.

Interpretation of algorithmic effectiveness was based on the findings reported within individual studies, considering experimental assumptions, baseline comparisons, evaluation metrics, and validation conditions rather than reporting-completeness scores. Claims of deployment readiness were considered separately in relation to validation realism, physical testing, and operational evidence. Consequently, reported performance improvements were interpreted within their study-specific settings and were not treated as proof of generalizable superiority or real-world readiness. Conceptual and framework-oriented studies were retained for contextual interpretation and research-gap identification rather than direct performance comparisons. Methods such as PSO, artificial bee colony optimization, whale optimization, crested porcupine optimization, glowworm swarm optimization, jellyfish optimization, genetic algorithms, and many-objective evolutionary algorithms are widely used for static placement, target coverage, connectivity, energy-aware deployment, and network-lifetime improvement [36,39,44,48,63,64,66,67,69,80]. Their main advantage is direct optimization of nonlinear and multi-objective deployment spaces, although validation remains predominantly simulation-based.

Reinforcement learning is less frequent but better suited to adaptive, sequential, and mobile deployment because placement or redeployment can be formulated as a decision process involving coverage, energy, topology, movement cost, and environmental change [31,34,45,65,71,76,77,79]. Its main limitations are sensitivity to reward design, state/action representation, training stability, and simulator realism.

Machine learning and deep learning approaches are more common in prediction-driven, localization-aware, and application-specific placement. ML methods support relationships between sensor position, environmental conditions, network behavior, and task-level performance [33,35,37,40,52,54,73,81]. Deep learning methods are particularly relevant when deployment quality depends on high-dimensional representation, reconstruction, recognition, topology inference, or prediction [37,58,62,65,67,74,75,78]. Their main challenges are data requirements, computational cost, and generalization across deployment conditions.

Hybrid approaches combine complementary techniques for multi-objective and application-specific deployment, including swarm-fuzzy optimization, DRL with graph learning, RL with differential evolution, Bayesian optimization with deep generative models, XAI-assisted search reduction, and centrality-guided RL [41,48,50,58,60,65,67,70,72,76,77,80]. Although promising, their added complexity requires stronger ablation studies and benchmarking. Bayesian and surrogate-assisted methods address expensive objective evaluation [32,70], while graph-based, topology-aware, centrality-based, and explainable-AI methods increasingly address network structure, observability, redundancy, and critical-node identification [42,58,72,74,76]. Conceptual and framework-oriented studies support interpretation and gap identification but should not be treated as direct evidence of algorithmic effectiveness [43,68].

The suitability of an AI method depends strongly on the underlying deployment problem structure. For static placement with continuous spatial decision variables and nonlinear coverage objectives, swarm-based and nature-inspired metaheuristics provide flexible search mechanisms. Genetic and evolutionary algorithms are particularly relevant to discrete or combinatorial decisions, such as sensor selection and candidate-location allocation, and can accommodate multiple competing objectives. Reinforcement learning is more appropriate when deployment involves sequential decisions, mobility, or changing network states, provided that meaningful state representations, reward functions, and training environments are available. Machine learning and deep learning support prediction-driven or task-specific placement when sufficient representative data exist, whereas graph-based methods are relevant when network topology, connectivity, or structural relationships influence deployment quality. Other problem characteristics further affect method selection. Bayesian and surrogate-assisted optimization may be useful when evaluating candidate placements is computationally expensive, while hybrid approaches can combine complementary capabilities for mixed-variable, multi-objective, or application-specific problems. However, these potential advantages must be weighed against computational complexity, data requirements, training stability, constraint handling, scalability, and deployment realism. Consequently, method selection should consider decision-variable type, objective formulation, environmental dynamics, topology dependence, evaluation cost, and available computational resources. As summarized in Table 11, the existing evidence supports these problem-dependent methodological tendencies rather than a universally superior AI family. These interpretations are qualitative and do not establish comparative superiority under standardized experimental conditions.

### 4.2. Discussion Based on Research Questions

#### 4.2.1. RQ1: What AI Techniques Are Used for Wireless Sensor Deployment Optimization?

The reviewed studies show that AI-driven WSN deployment employs two broad groups of techniques: optimization-based approaches, including evolutionary and swarm-based metaheuristics, and learning-based approaches, including machine learning, deep learning, and reinforcement learning. Supporting techniques such as fuzzy logic and graph-based learning are frequently integrated with these methods, while hybrid approaches combine complementary components to address complex deployment objectives. Because these categories overlap, their frequencies indicate methodological prevalence rather than mutually exclusive distributions. This pattern reflects the constrained and multi-objective nature of sensor deployment, where placement decisions must balance coverage, connectivity, reliability, energy consumption, cost, redundancy, localization accuracy, and computational burden.

Swarm-intelligence and nature-inspired metaheuristics form the largest algorithmic group. PSO-based approaches are widely used for coverage, connectivity, target monitoring, and energy-aware placement [39,44,48,64], while artificial bee colony, whale, crested porcupine, glowworm swarm, jellyfish, and related methods address similar nonlinear and multi-objective deployment problems [55,57,63,66,67,69]. Evolutionary methods, including genetic and many-objective algorithms, are also used for coverage-hole detection, combinatorial placement, wearable sensing, 3D deployment, and joint optimization of coverage, connectivity, cost, and load balancing [36,53,56,80]. These methods are flexible for complex search spaces, although their evidence remains largely simulation-based and sensitive to modeling assumptions.

ML and DL methods are primarily used for prediction-supported and application-specific placement. ML supports obstruction-aware planning, localization-aware deployment, redundancy reduction, smart-home sensing, drainage monitoring, dynamic placement, and smart-city applications by learning relationships among sensor locations, environmental conditions, network behavior, and task performance [33,35,37,40,52,54,73,81]. DL extends this capability to high-dimensional and task-specific settings such as structural reconstruction, activity recognition, sparse inertial sensing, topology identification, dependability planning, and coverage control [58,62,65,67,74,75,78]. Its main limitations are training-data requirements, computational cost, and the need for stronger generalization testing.

RL and deep RL are less common than metaheuristics but are particularly suited to adaptive, mobile, and sequential deployment. These approaches formulate placement or redeployment as a decision process driven by coverage, energy, topology, movement cost, and other network states [31,34,45,65,71,76,77,79]. Their effectiveness, however, depends strongly on reward design, state and action representation, training conditions, and simulator realism.

Hybrid methods increasingly combine complementary techniques for multi-objective deployment, including swarm-fuzzy optimization, RL with differential evolution, DRL with graph neural networks, Bayesian optimization with deep generative models, XAI-assisted search reduction, and centrality-guided RL [41,48,50,58,60,65,67,70,72,76,77,80]. These approaches are promising for complex deployment problems but require stronger ablation studies and benchmarking to justify their added complexity.

Overall, AI-driven WSN deployment has progressed toward adaptive, topology-aware, and application-specific optimization. Method selection should be guided by the structure of the deployment problem rather than algorithmic popularity alone. Static spatial optimization, discrete sensor selection, sequential redeployment, data-driven prediction, and topology-aware placement impose different methodological requirements. However, inconsistent benchmarking and validation continue to limit reliable comparisons of method suitability across deployment settings.

#### 4.2.2. RQ2: What Deployment Objectives Are Addressed in the Selected Studies?

Coverage maximization is the most frequently reported deployment objective, appearing in 31 of the 51 included studies. It is addressed across classical WSN deployment, mobile redeployment, target coverage, underwater sensing, 3D deployment, and adaptive coverage control. However, this frequency includes studies where coverage is considered alongside connectivity, energy efficiency, network lifetime, or other objectives. Thus, the reported count indicates the prevalence of coverage-related optimization rather than establishing that coverage was the primary objective in all 31 studies. Its dominance reflects the fundamental requirement that deployed sensors adequately observe the intended area, targets, or events.

Connectivity preservation is another major objective because sensing coverage alone does not ensure that collected data can reach a sink or processing unit. Studies therefore evaluate connectivity, packet delivery, topology robustness, communication feasibility, and network stability, particularly in large-scale, heterogeneous, underwater, and dynamic networks [31,34,37,43,48,56,60,63,64,67,76,80].

Energy efficiency and network lifetime also form a dominant objective cluster. Deployment strategies aim to reduce unnecessary movement, redundant sensing, communication overhead, and uneven energy depletion [31,48,57,58,61,69,73,76,78,81]. Load balancing and residual-energy preservation are also considered because uneven depletion can create coverage or connectivity failures [48,56].

Cost reduction and sensor-count minimization are important where devices or installation are expensive. These objectives appear in WSN optimization, smart-home and wearable sensing, structural monitoring, water systems, pipeline monitoring, and 3D IoT deployment [38,45,53,59,60,70,72,80]. The goal is to maintain adequate sensing performance while reducing deployment effort and hardware requirements.

Other studies emphasize localization accuracy and task-level information quality rather than geometric coverage alone. Metrics include localization error, position estimation, topology observability, damage-detection accuracy, reconstruction error, activity-recognition accuracy, and prediction accuracy [37,49,50,54,59,62]. These objectives are particularly relevant when deployment supports downstream inference or diagnostic tasks.

Robustness, dependability, fault tolerance, latency, and adaptability receive comparatively less attention. Existing studies consider failure tolerance, network stability, overlap reduction, delay, packet delivery, failure probability, and dependability [38,42,44,48,49,60,67,75,80,81]. However, these objectives remain less consistently addressed than coverage, connectivity, and energy, despite their importance under node failure, interference, battery aging, environmental uncertainty, and changing sensing requirements.

#### 4.2.3. RQ3: What WSN Environments and Application Domains Are Considered?

The reviewed studies cover both generic WSN models and domain-specific intelligent sensing systems. A substantial portion of the literature still relies on abstract 2D or static WSN environments for algorithmic evaluation [31,36,39,42,44,48,49,55,56,63,64,66,76,78,81]. These settings support controlled comparison but often simplify terrain, obstacles, sensing uncertainty, radio propagation, hardware limitations, and localization error.

Three-dimensional deployment is represented in 3D WSNs, IoT smart spaces, directional sensing, and structural or spatial monitoring [41,45,46,51,58,67,68,80]. Such environments introduce additional challenges involving volumetric coverage, sensing orientation, obstacle interaction, and connectivity, limiting the direct transfer of methods validated only in 2D settings.

Mobile and adaptive deployment is addressed through reinforcement learning, redeployment, adaptive coverage control, and hybrid optimization [31,34,50,58,61,65,69,76,77,79]. These approaches are relevant when node mobility, changing event regions, environmental variation, or failures require placement to be updated over time. Underwater and aquatic networks represent another specialized setting, where deployment must account for acoustic communication, limited bandwidth, high energy consumption, and difficult maintenance [57,69].

Application-specific studies further extend deployment beyond geometric coverage. Smart-home, healthcare, wearable, activity-recognition, and assisted-living systems connect placement to recognition accuracy, posture reconstruction, plantar-pressure estimation, unobtrusiveness, user behavior, and privacy [40,47,53,54,59,62]. Industrial IoT, structural health monitoring, pipeline systems, and active distribution networks instead emphasize diagnosis, observability, damage detection, reconstruction, and production monitoring [37,43,46,51,70,72,74,79].

Smart-city, transportation, urban-drainage, and dependability-oriented studies further broaden the evidence base toward large-scale planning, uncertainty, changing demand, and safety-critical sensing [35,45,52,55,75]. Overall, AI-driven WSN deployment spans diverse environments, and the meaning of “optimal placement” depends strongly on the sensing task, physical environment, data pathway, and operational constraints. Recent practical applications further illustrate the importance of environment-aware deployment planning. He et al. [83] investigated UAV trajectory and resource optimization for secure low-altitude communications under mixed obstacles, using multi-agent deep reinforcement learning to address three-dimensional flight restrictions, energy limitations, and communication security. In traffic wireless sensor networks, Zhang et al. [84] proposed RoadNet, a graph-based relay deployment approach that considers road topology and sensor coverage while reducing deployment cost and computational overhead. Although these studies address related communication and relay-planning problems rather than the specific AI-driven sensor-placement scope of this review, they illustrate practical requirements involving spatial constraints, network topology, energy availability, security, and infrastructure-aware planning.

#### 4.2.4. RQ4: What Performance Metrics Are Used to Evaluate AI-Driven Deployment Methods?

The most common evaluation metrics are coverage ratio, connectivity, energy consumption, residual energy, network lifetime, node count, movement distance, deployment cost, convergence speed, computational time, packet delivery ratio, throughput, delay, and localization error. Coverage ratio is the most frequently reported metric because it directly measures whether the intended sensing region or target set is adequately monitored [31,32,34,36,39,44,48,49,69,76,78,80].

Energy-related metrics include total energy consumption, residual energy, energy efficiency, movement energy, and network lifetime [31,48,57,58,61,69,73,76,78,81]. These measures capture the trade-off between improved sensing performance and the additional communication, movement, or processing costs that may shorten network lifetime.

Connectivity and communication are commonly evaluated using connectivity ratio, packet delivery ratio, throughput, delay, path reliability, and network stability [31,34,37,43,48,60,63,64,67,75,76,80,81]. These metrics are particularly important in multi-hop and large-scale WSNs, where adequate sensing coverage does not guarantee reliable data delivery.

Application-specific studies additionally use task-level metrics such as localization and reconstruction error, recognition and prediction accuracy, topology-identification accuracy, damage-location and severity accuracy, detection time, dependability, and observability [37,49,50,59,62]. Such metrics reflect deployment quality when the network supports inference, diagnosis, or other downstream analytical tasks rather than spatial coverage alone.

Despite this diversity, metric inconsistency remains a major methodological limitation. Studies differ in coverage definitions, sensing and communication models, obstacle assumptions, energy models, node densities, deployment areas, and baseline algorithms [39,44,48,69,70,76,80]. Consequently, reported improvements in coverage, energy, or lifetime are often not directly comparable across studies.

Future evaluations should therefore use more standardized metric sets. At minimum, deployment studies should report coverage, connectivity, energy consumption, network lifetime, computational cost, baseline configuration, sensing and communication models, node assumptions, and robustness conditions. Application-specific studies should additionally report relevant task-level measures such as recognition accuracy, reconstruction error, detection accuracy, or observability.

#### 4.2.5. RQ5: What Validation Methods, Simulators, Datasets, and Baselines Are Used?

Simulation is the dominant validation method across reinforcement learning, swarm and evolutionary optimization, Bayesian optimization, virtual-force deployment, and hybrid approaches [31,32,34,39,41,44,48,50,61,69]. Simulation enables controlled and repeatable evaluation of node density, sensing and communication ranges, deployment area, mobility, and optimization parameters while avoiding the cost of physical experiments.

However, simulation dependence remains the main validation weakness. Many studies assume ideal sensing, stable communication, accurate localization, simplified mobility, uniform terrain, and abstract energy models, whereas real deployments involve radio irregularity, fading, obstacles, interference, battery aging, sensor drift, hardware failure, and maintenance constraints. Simulation-only results should therefore be interpreted primarily as evidence of algorithmic feasibility rather than deployment readiness.

Dataset-based and application-specific validation is more common in machine learning and deep learning studies, including wearable sensing, smart homes, structural monitoring, water systems, spatiotemporal modeling, and distribution networks [40,46,47,51,59,60,62]. These evaluations provide stronger task-level evidence by linking placement to real or realistic data, although their generalizability may be limited by dataset size, scenario coverage, subject diversity, sensor modality, and transferability.

Numerical and structural models introduce application-specific physical assumptions beyond generic computational evaluation, although their realism depends on model calibration, assumptions, and correspondence with the intended deployment environment, and they do not substitute for operational testing.

Baseline selection is also inconsistent. Some studies compare against established methods such as PSO, GA, DE, ACO, exact optimization, virtual-force approaches, or multiple state-of-the-art metaheuristics [69,70,76,80], whereas others use limited baselines or report mainly internal performance. This inconsistency limits confidence in cross-study claims of superiority.

Overall, validation evidence remains uneven across the reviewed studies, particularly with respect to physical evaluation, operational deployment, reproducibility, and long-term observation. Stronger studies clearly define the deployment problem, report objectives and metrics, use meaningful baselines, and describe their evaluation settings, whereas weaker studies provide insufficient detail on assumptions, parameters, limitations, or reproducibility. Future research should therefore emphasize shared benchmarks, reproducible experimental artifacts, digital twins, physical testbeds, and long-term field validation.

#### 4.2.6. RQ6: What Are the Major Limitations, Challenges, and Future Research Directions?

A major challenge is the lack of a unified, realistic, and reproducible framework for AI-driven WSN deployment. The literature remains fragmented across algorithm families, objectives, application domains, validation settings, and performance metrics. While coverage and energy are well studied, robustness, uncertainty, latency, security, privacy, edge feasibility, lifecycle maintenance, and real-world transferability receive comparatively limited attention.

Another challenge is the limited integration of deployment, localization, communication, and downstream analytics. Localization-aware studies show that position uncertainty affects deployment quality [37,49,50], while application-specific studies demonstrate that placement influences activity recognition, posture reconstruction, topology identification, damage detection, energy forecasting, and smart-city dependability [40,46,47,51,53,54,59,62]. However, few approaches jointly optimize placement, localization accuracy, communication reliability, energy use, and task-level performance.

Real-world transferability also remains limited. Many methods are validated under controlled simulation assumptions and are not tested against obstacles, interference, battery aging, sensor drift, hardware failures, changing monitoring requirements, or maintenance constraints [31,32,34,39,44,48,69]. Application-specific studies provide stronger practical evidence but are often restricted to narrow scenarios, limited datasets, or domain-specific conditions [59,62].

Benchmarking is another major weakness. Differences in deployment scenarios, node assumptions, sensing and communication models, energy models, and baseline algorithms make fair comparison across AI families difficult. This is especially important for hybrid methods, whose additional complexity should be justified through ablation studies, reproducibility, and consistent performance across multiple scenarios [41,48,50,58,60,65,67,70,72,76,77,80].

Future research should prioritize integrated multi-objective deployment, uncertainty-aware placement, realistic 3D and obstacle-aware modeling, adaptive redeployment, edge-compatible and interpretable AI, standardized benchmarks, and long-term validation. Deployment should be treated as a lifecycle problem in which sensing quality, connectivity, energy balance, localization accuracy, robustness, security, privacy, and application-level performance are maintained beyond the initial placement stage.

### 4.3. Implications for Sensor Data Analysis and Intelligent Processing

The deployment patterns identified in the reviewed studies have important implications for downstream sensor-data analysis and intelligent processing. This subsection interprets those implications from the reviewed evidence; unless explicitly supported by cited studies, they should be understood as methodological considerations rather than empirical findings uniformly extracted from the corpus. Sensor placement affects not only network coverage and connectivity but also which spatial regions, events, activities, or system states are represented in the collected data. Application-specific studies illustrate this connection in smart-home activity recognition, structural monitoring, topology identification, reconstruction, and related sensing tasks.

Coverage limitations may therefore lead to under-representation of relevant regions or operating conditions in the resulting data. Rather than assuming a particular statistical missingness mechanism, a more cautious interpretation is that insufficient spatial or task coverage can create structurally missing or weakly represented observations relative to the intended sensing process. Conversely, dense deployments may produce highly redundant measurements while increasing communication, storage, and energy costs. Deployment design should therefore balance coverage, redundancy, and information value according to the application and sensing objective.

Sensor placement can also influence the effectiveness of multi-sensor fusion. Fusion methods may benefit from complementary spatial, temporal, or modal observations, but the degree of independence required depends on the specific fusion model and should not be treated as a universal assumption. Poorly distributed sensors may provide highly similar observations while failing to capture informative spatial perspectives. This consideration is particularly relevant in application-specific settings where task performance depends on sensing informative locations, as observed in activity-recognition and structural-reconstruction studies.

Similar considerations apply to anomaly detection, calibration, and fault diagnosis. Sensors located near informative, high-risk, or transition regions may provide more useful evidence for detecting abnormal behavior, whereas spatial redundancy can support cross-checking, fault identification, and drift handling. However, additional redundancy can also increase energy and maintenance costs. The appropriate balance is therefore application-dependent rather than universally defined.

Deployment also has implications for edge-AI operation and model transferability. For online or edge-executed methods, relevant constraints include local computation, communication overhead, energy consumption, and hardware limitations, whereas these constraints are less restrictive for one-time offline deployment planning. The reviewed literature also indicates that application-specific results may depend on particular layouts, participants, sensor modalities, or operating conditions, which can limit transferability across settings. Accordingly, differences between development and deployment environments may affect downstream model performance when spatial coverage, sensing density, or the resulting data distribution changes.

Overall, these observations suggest that AI-driven sensor deployment should be considered not only as a geometric coverage problem but also as part of the broader data-generation and processing pipeline. Future deployment studies should therefore report how placement decisions interact with data representativeness, redundancy, task-level sensing quality, computational requirements, and application-specific analytical objectives.

### 4.4. Benchmarking Requirements for AI-Driven WSN Deployment

A major barrier to comparing AI-driven WSN deployment studies is the lack of standardized benchmarks. Studies differ in deployment areas, node densities, sensing and communication models, energy assumptions, objective functions, and baselines, making reported improvements in coverage, connectivity, energy, or localization difficult to compare directly. Performance obtained in an idealized 2D environment may not transfer to obstacle-rich, 3D, mobile, heterogeneous, or energy-constrained settings.

A benchmarking framework should therefore define common deployment scenarios, node assumptions, sensing and communication models, evaluation metrics, baselines, and reproducibility requirements. Such a framework would complement rather than replace application-specific evaluation by providing a shared layer for comparing metaheuristics, machine learning, deep learning, reinforcement learning, Bayesian optimization, graph-based methods, and hybrid approaches. Benchmarks should include both controlled scenarios for algorithmic comparison and progressively realistic settings incorporating obstacles, communication degradation, localization noise, mobility, heterogeneous nodes, energy depletion, and failures.

Benchmark studies should clearly specify sensing and communication assumptions. Binary sensing and fixed communication radii may be appropriate for early-stage algorithmic testing, but realistic evaluation should progressively include probabilistic or directional sensing, obstruction effects, path loss, RSSI variation, interference, packet loss, and multi-hop communication.

Evaluation metrics should also be standardized beyond coverage alone. Relevant measures include connectivity, energy consumption, network lifetime, localization error, packet delivery ratio, latency, computational cost, deployment cost, and robustness. Lifetime definitions should distinguish measures such as first-node death, half-node death, last-node death, connectivity lifetime, or coverage-preserving lifetime. Localization studies should specify the error measure used, while robustness should be evaluated under conditions such as node failure, sensing noise, localization error, or communication degradation.

The benchmarking requirements in Table 12 represent a proposed evaluation specification derived from recurring comparability gaps in the reviewed literature. They are intended to guide future benchmark development rather than constitute an implemented benchmark suite (Table 13). Accordingly, this review does not provide fixed benchmark instances, executable scenario generators, reference algorithm implementations, or experimentally validated benchmark results. These elements require separate implementation and validation before standardized cross-method comparisons can be performed.

Reproducibility should be a basic benchmarking requirement. Studies should report parameter settings, random seeds, node coordinates or scenario-generation rules, simulation configurations, baseline implementations, and source code where possible. Dataset-based studies should additionally document data sources, preprocessing, train-test splits, validation procedures, and evaluation scripts.

A practical benchmark should distinguish a common reporting core from application-specific modules. The common core should specify deployment geometry and units, node number or density, sensing and communication assumptions, objective functions and constraints, baseline methods, parameter ranges, computational or function-evaluation budgets, development and held-out test scenarios, repeated random seeds, and reporting statistics. Application-specific modules should add only relevant requirements, such as acoustic propagation for underwater networks, body-location constraints for wearable sensing, directional sensing or occlusion where appropriate, or mobility constraints for UAV-assisted systems. Consequently, all application domains need not use identical metrics or scenarios. Practical applications involving obstacle-constrained UAV communications and road-network-aware relay placement [83,84] further motivate application-specific benchmarking of geometric constraints, communication reliability, topology, energy budgets, and deployment cost.

## 5. Critical Analysis

### 5.1. Strengths of Existing Studies

The literature shows a clear progression from conventional coverage-driven deployment toward adaptive, intelligent, and application-aware sensor placement. Classical optimization, swarm intelligence, and evolutionary methods provide a strong foundation by formulating deployment as a multi-objective problem involving coverage, connectivity, energy consumption, network lifetime, cost, redundancy, robustness, and load balancing. PSO variants, genetic algorithms, artificial bee colony optimization, whale optimization, crested porcupine optimization, glowworm swarm optimization, jellyfish optimization, and many-objective evolutionary methods demonstrate the continued effectiveness of population-based search for complex spatial deployment problems [36,39,44,48,63,64,66,67,69,80]. These studies establish useful algorithmic baselines for coverage improvement, energy-aware placement, blind-spot reduction, and lifetime extension.

A major strength is the increasing treatment of deployment as an adaptive rather than one-time decision problem. Reinforcement learning, deep reinforcement learning, hybrid RL–DE optimization, graph-assisted DRL, centrality-guided RL, and related approaches support sequential placement, redeployment, and sensor-allocation decisions under changing network conditions [31,34,45,65,71,76,77,79]. These methods are particularly relevant when coverage demand, energy availability, node failures, mobility, or environmental conditions evolve during operation.

Machine learning and deep learning studies further extend deployment toward data-driven and task-aware sensing. These approaches support localization-aware placement, obstruction-aware planning, redundancy identification, smart-home sensing, activity recognition, posture reconstruction, topology identification, dependability analysis, and dynamic placement prediction [33,35,37,40,46,47,62,78,81]. Their main contribution is the use of task-level outcomes such as localization error, recognition accuracy, reconstruction error, topology observability, dependability, and prediction accuracy rather than geometric coverage alone.

Another strength is the growing integration of localization and topology information into deployment decisions. Localization-aware optimization demonstrates that placement quality depends on position uncertainty [37,49,50], while graph-based, centrality-based, and explainable-AI methods incorporate structural importance, topology contribution, observability, redundancy, and critical-node identification [42,58,72,76]. This moves the field beyond coordinate-only placement toward structurally informed sensing design.

The literature has also expanded beyond simplified two-dimensional and homogeneous environments. Three-dimensional deployment, directional sensing, underwater WSNs, roadside sensing, smart spaces, structural monitoring, active distribution networks, and pipeline systems introduce constraints related to geometry, orientation, mobility, communication, candidate locations, and harsh operating conditions [41,45,46,51,57,74,80]. These studies strengthen the practical relevance of AI-driven deployment by considering increasingly realistic and domain-specific environments.

Application-oriented research provides further evidence that optimal placement depends on the sensing task and operational context. Smart-home monitoring, activity recognition, wearable sensing, posture reconstruction, structural health monitoring, water systems, smart-city dependability, and IIoT applications link deployment to user requirements, maintenance, privacy, power constraints, and downstream analytics [40,47,59,60,62,70,72,75,79]. These studies demonstrate that effective placement should improve information and decision quality rather than only maximize spatial coverage.

Finally, hybrid methods are increasingly used to address the multi-objective nature of deployment by combining swarm optimization, fuzzy logic, deep learning, graph learning, reinforcement learning, Bayesian optimization, explainable AI, and centrality analysis [48,58,60,65,67,70,72,76,77]. By integrating global search, adaptation, prediction, topology awareness, and interpretability, these approaches offer a promising direction toward more realistic deployment pipelines when supported by rigorous validation.

### 5.2. Weaknesses of Existing Studies

Despite these strengths, the literature remains heavily dependent on simulation-based validation. Many deployment methods report improvements in coverage, convergence, energy efficiency, lifetime, or localization accuracy under simplified assumptions such as ideal sensing and communication ranges, controlled node distributions, and limited environmental uncertainty [31,32,34,39,44,48,61,69]. Real deployments, however, are affected by fading, obstacles, packet loss, battery degradation, node failure, localization drift, interference, terrain variation, installation constraints, and hardware imperfections. Simulation-only evidence therefore provides limited assurance of real-world transferability.

A second weakness is the narrow treatment of deployment objectives. Coverage-centered methods often simplify routing overhead, communication reliability, localization uncertainty, latency, security, and long-term energy imbalance [36,39,44,48,55,63,66,67]. Energy- and lifetime-aware studies address some of these concerns but frequently rely on abstract energy models that omit sensing, processing, retransmission, mobility, edge-inference, and maintenance costs [57,61,64,65,69]. Consequently, reported gains may reflect performance under controlled assumptions rather than complete operational behavior.

The literature is also fragmented across deployment, localization, communication, routing, and analytics. Localization-aware studies improve position estimation but do not always integrate localization uncertainty with coverage repair, routing, or energy-aware redeployment [37,49,50]. Likewise, ML- and DL-based studies improve prediction, recognition, reconstruction, topology identification, or dependability without consistently optimizing communication reliability and energy use [46,47,62,70,74,75]. Although cross-layer and application-specific approaches provide progress, integrated end-to-end deployment optimization remains limited [37,72,79].

Application-specific studies often provide stronger practical evidence but limited generalizability. Smart-home, wearable, activity-recognition, plantar-pressure, and senior-monitoring results may depend on particular participants, layouts, sensor types, body locations, activities, or deployment durations [40,47,53,54,59,62]. Structural, pipeline, distribution-network, and spatiotemporal studies are similarly tied to specific structures, network models, or finite-element environments [46,51,74]. These constraints limit cross-domain transferability.

Another major weakness is poor cross-study comparability. Reported improvements are obtained using different deployment areas, node densities, sensing and communication models, energy assumptions, objective functions, and baselines. Consequently, results from swarm-based, DRL-GNN, 3D IoT, or underwater deployment studies cannot be directly compared unless their experimental assumptions are aligned [69,76,80]. The current evidence therefore supports within-study comparison and cross-method trend analysis, but not statistically reliable pooled ranking across AI families.

Baseline selection and reproducibility are also inconsistent. Some studies compare against established methods such as PSO, GA, DE, ACO, virtual-force approaches, exact optimization, or multiple state-of-the-art metaheuristics [69,70,76,80], whereas others use limited baselines or mainly report internal improvements. Many studies also provide insufficient detail on random seeds, parameter settings, sensing and communication models, code, datasets, deployment maps, or hardware assumptions. This is particularly problematic for hybrid and deep learning methods, whose performance depends strongly on architecture, training data, reward design, hyperparameters, and ablation settings [58,65,67,70,72,76,77,78]. These limitations make algorithmic claims difficult to reproduce and verify.

Finally, conceptual, review-oriented, and framework-level studies are valuable for identifying trends, methodological gaps, and future directions, but they should not be treated as direct evidence of algorithmic effectiveness [43,68]. Their role is primarily interpretive rather than experimental.

### 5.3. Research Gap

Several recurring gaps emerge from the reviewed literature. First, most studies optimize only subsets of deployment requirements rather than jointly addressing coverage, connectivity, localization uncertainty, energy, reliability, robustness, security, privacy, computational cost, and application-specific sensing quality. Swarm and evolutionary methods mainly emphasize coverage and connectivity [39,44,48,55,56,63,64,66,67], reinforcement learning supports adaptive deployment [31,34,45,65,71,76,77,79], and localization-aware methods address position uncertainty [37,49,50].

Second, realistic deployment conditions remain underrepresented. Many studies rely on simplified 2D settings, while practical systems involve obstacles, mobility, communication constraints, 3D geometry, installation restrictions, and application-specific environments [45,52,57,79,80].

Third, standardized benchmarking is limited. Differences in datasets, node assumptions, sensing and communication models, metrics, baselines, and evaluation settings restrict fair cross-method comparison. Future benchmarks should therefore use reproducible scenarios, clearly defined assumptions, repeated runs, and comparable evaluation protocols.

Fourth, edge feasibility requires role-specific interpretation. Offline planners should not be judged by the same latency, memory, and energy constraints as online or on-node policies. Relevant factors for online methods include execution location, decision frequency, compute and memory requirements, training and inference cost, energy use, and communication overhead. These variables were not systematically coded in this review and are therefore treated as future reporting requirements rather than study-level feasibility scores.

Fifth, lifecycle adaptation remains limited. Most studies focus on initial placement despite battery depletion, sensor drift, node failure, environmental change, and evolving monitoring requirements [52,58,61,65,69,70,75,79]. Adaptive redeployment, recalibration, self-healing coverage, and maintenance-aware optimization remain underdeveloped.

Finally, task-level performance is not consistently integrated into deployment optimization. Future methods should connect physical placement with downstream objectives such as recognition, reconstruction, diagnosis, topology identification, contamination detection, and dependability rather than relying primarily on geometric coverage.

### 5.4. Proposed Conceptual and Evidence-Informed AI–Deployment–Validation Lifecycle Framework

Based on recurring patterns identified through the systematic mapping, cross-method comparison, and critical synthesis, this review proposes a conceptual and evidence-informed AI–Deployment–Validation Lifecycle Framework for AI-driven WSN deployment research (Table 14). The framework organizes the progression from application definition and objective formulation to AI-method selection, deployment optimization, validation, benchmarking, and lifecycle adaptation. Its stages reflect recurring gaps in the 51-study evidence corpus, including fragmented objectives, simplified environments, simulation-dominated validation, weak benchmarking and reproducibility, and limited lifecycle adaptation.

The framework has not been empirically tested, independently validated by external experts, or applied as a formal scoring or maturity-assessment instrument. It should therefore be interpreted as a synthesis-oriented framework for organizing future research rather than as a validated model for ranking individual studies. Each stage is linked to recurring gaps observed in the coded corpus; the cited studies provide representative evidence for those gaps rather than empirical validation of the framework itself.

### 5.5. Future Research Directions

Future work should operationalize the proposed framework into explicit qualitative indicators for each lifecycle stage. A subsequent study could retrospectively apply these indicators to a representative sample of studies from swarm intelligence, evolutionary optimization, reinforcement learning, machine learning, deep learning, Bayesian optimization, graph-based learning, and hybrid methods. Such an application could examine which lifecycle stages are addressed, partially addressed, or absent, followed by independent expert review and inter-rater agreement analysis. This would determine whether the framework can be developed from a conceptual synthesis into a reliable appraisal or design-support instrument.

Another important direction is localization-aware deployment under uncertainty. Future models should not assume exact node coordinates. Instead, localization error should be included directly in the deployment objective through uncertainty-aware coverage estimation, average localization error prediction, range-free localization, virtual-anchor methods, and probabilistic node-position modeling [37,49,50]. This is especially important for GPS-denied, mobile, underwater, indoor, industrial, and disaster-response environments, where localization error can degrade both coverage and routing decisions.

Research should also prioritize realistic 3D and environment-aware deployment. Directional sensor networks, 3D WSNs, smart-space IoT deployment, roadside sensing, underwater monitoring, structural health monitoring, and pipeline systems demonstrate that orientation, occlusion, geometry, candidate locations, communication medium, and information value matter as much as node density [41,45,57,80]. Future studies should model deployment as a constrained spatial-intelligence problem rather than a simplified coverage-maximization task. This includes incorporating digital twins, building information models, terrain maps, RF propagation models, acoustic propagation models, finite-element models, hydraulic models, and real-time environmental feedback.

A further direction is standardized and reproducible benchmarking for AI-driven deployment. Benchmarks should compare classical algorithms, swarm intelligence, evolutionary algorithms, supervised machine learning, deep learning, reinforcement learning, Bayesian optimization, graph neural networks, XAI-assisted placement, and hybrid methods under common assumptions. Metrics should include coverage ratio, connectivity robustness, localization error, energy lifetime, communication overhead, computational cost, deployment cost, fault tolerance, latency, dependability, task-level accuracy, and real-world transferability. Without shared benchmarks, claims of superiority across studies will remain difficult to interpret. Future work should operationalize the proposed scenarios into fixed benchmark instances with specified network dimensions, node configurations, sensing and communication parameters, evaluation budgets, and random seeds. Open-source reference implementations of established baseline algorithms, together with standardized evaluation scripts and documented configurations, would enable reproducible and fair comparisons across AI-driven deployment methods.

Future research should strengthen real-world validation and edge implementation. Smart-home, wearable, senior-housing, IIoT, structural-monitoring, smart-city, and pipeline studies show that practical deployment is shaped by usability, privacy, maintenance, calibration, power, reliability, and user trust [40,47,59,62,70,72,75,79]. Deployment systems should therefore be tested in physical environments over longer periods, with real sensors and realistic communication constraints. At the same time, ML and DL models should be compressed, explainable, privacy-preserving, and suitable for embedded or edge execution [78].

Finally, future work should treat sensor deployment as a lifecycle problem. Initial placement is only the first stage. Practical networks require recalibration, sensor replacement, adaptive sleep scheduling, self-healing coverage, redeployment after node failure, model updating, and maintenance planning. The most impactful future work will not merely propose another optimizer; it will demonstrate a deployable system that remains reliable under localization uncertainty, environmental change, heterogeneous hardware, energy limits, privacy constraints, and long-term operational degradation.

## 6. Limitations of This Study

Although this review followed a PRISMA-guided systematic process, several limitations remain. First, the search may not have captured all relevant studies due to differences in database coverage, indexing, metadata quality, and platform functionality. Despite searching Scopus, Web of Science, IEEE Xplore, ACM Digital Library, ScienceDirect, SpringerLink, and MDPI, relevant work may exist in unindexed venues or institutional repositories [41,43]. Restricting the review to English-language publications may also have excluded research from other regions.

The relatively limited initial retrieval count of 124 records also reflects the focused search strategy, which combined WSN-related, deployment-related, and AI-related terms. This approach may have missed relevant studies using alternative terminology or indexing conventions. Therefore, the final corpus of 51 studies represents a focused synthesis of the retrieved literature rather than an exhaustive account of all AI-driven WSN deployment research.

Second, the search was sensitive to terminology. Despite including major WSN, deployment, optimization, and AI-related terms, relevant studies may use alternatives such as “sensor scheduling,” “topology control,” “coverage control,” “self-organization,” “node relocation,” or “placement planning.” Such variations are common across networking, optimization, computing, and application domains [43,46,47].

Third, quantitative meta-analysis was inappropriate due to substantial heterogeneity in deployment areas, node densities, sensing and communication models, energy assumptions, objectives, simulators, datasets, baselines, and evaluation metrics. Reported outcomes included coverage, connectivity, localization error, energy consumption, lifetime, cost, reconstruction error, recognition accuracy, and computational time. Without common effect sizes or standardized benchmarks, statistical pooling would be unreliable [44]. Moreover, performance improvements reported under different experimental conditions cannot be directly compared. Accordingly, the review employed descriptive statistics, systematic mapping, cross-method comparison, and narrative synthesis rather than pooled performance rankings.

Fourth, the study-level appraisal assessed the completeness of methodological reporting rather than comprehensive methodological validity or risk of bias. The checklist did not directly evaluate baseline-tuning fairness, statistical uncertainty, robustness, train–test leakage, hyperparameter-selection bias, external validity, reproducibility, or the realism of sensing and communication assumptions. Although considered qualitatively, these aspects require a dedicated risk-of-bias instrument for AI-driven WSN deployment. Therefore, reporting-completeness scores should not be interpreted as indicators of algorithmic reliability, comparative superiority, or operational deployment readiness. Establishing these properties requires direct assessment of methodological validity, potential bias, and external validation evidence.

Fifth, recent, early-access, or in-press studies may have been missed because the field is rapidly evolving, particularly in deep reinforcement learning, graph neural networks, edge AI, digital twins, UAV-assisted sensing, and autonomous placement. Thus, the findings represent a systematic snapshot of the literature within the defined search period rather than a permanent assessment of the field.

Finally, the proposed AI–Deployment–Validation Lifecycle Framework is conceptual and evidence-informed, derived from recurring patterns and gaps identified through systematic mapping and narrative synthesis. It has not been independently validated through expert elicitation, Delphi analysis, empirical implementation, or retrospective scoring. Its stages therefore serve as organizing dimensions rather than validated measurement constructs. Future research should assess its completeness, practical usability, and reliability across AI methods, WSN environments, and validation settings.

## 7. Conclusions

This PRISMA-guided systematic review and mapping study examined 51 studies on AI-driven wireless sensor network deployment. The review focused on how artificial intelligence has been applied to sensor placement, redeployment, coverage optimization, connectivity preservation, energy-aware deployment, localization-aware deployment, and application-specific sensing.

The findings show that optimization-oriented AI methods, especially metaheuristics and swarm-intelligence algorithms, dominate the field because WSN deployment is naturally a constrained search and multi-objective optimization problem. Machine learning, deep learning, and reinforcement learning are emerging in deployment scenarios that involve prediction, environment modeling, adaptive redeployment, mobile sensing, sensor ranking, and energy forecasting. Across the reviewed studies, coverage, connectivity, energy efficiency, and network lifetime are among the most frequently represented objectives in the non-mutually exclusive mapping.

The main methodological weakness of the field is validation. A large proportion of studies still depend on simulation-only evaluation, simplified sensing and communication assumptions, idealized energy models, limited mobility settings, and non-standardized baselines. As a result, direct comparison across studies remains difficult, and the practical transferability of many proposed methods is still uncertain.

This review contributes by moving the discussion beyond algorithm classification toward evidence quality, validation maturity, benchmarking, reproducibility, and deployment readiness. It proposes a conceptual and evidence-informed AI–Deployment–Validation Lifecycle Framework as an organizing structure for future research. The framework has not yet been empirically or independently validated and should not be interpreted as a formal scoring instrument. Its present contribution is to integrate recurring application, optimization, validation, benchmarking, and lifecycle requirements into a coherent research pipeline. Future work should operationalize and evaluate the framework across representative studies and deployment settings.

## Figures and Tables

**Figure 1:**
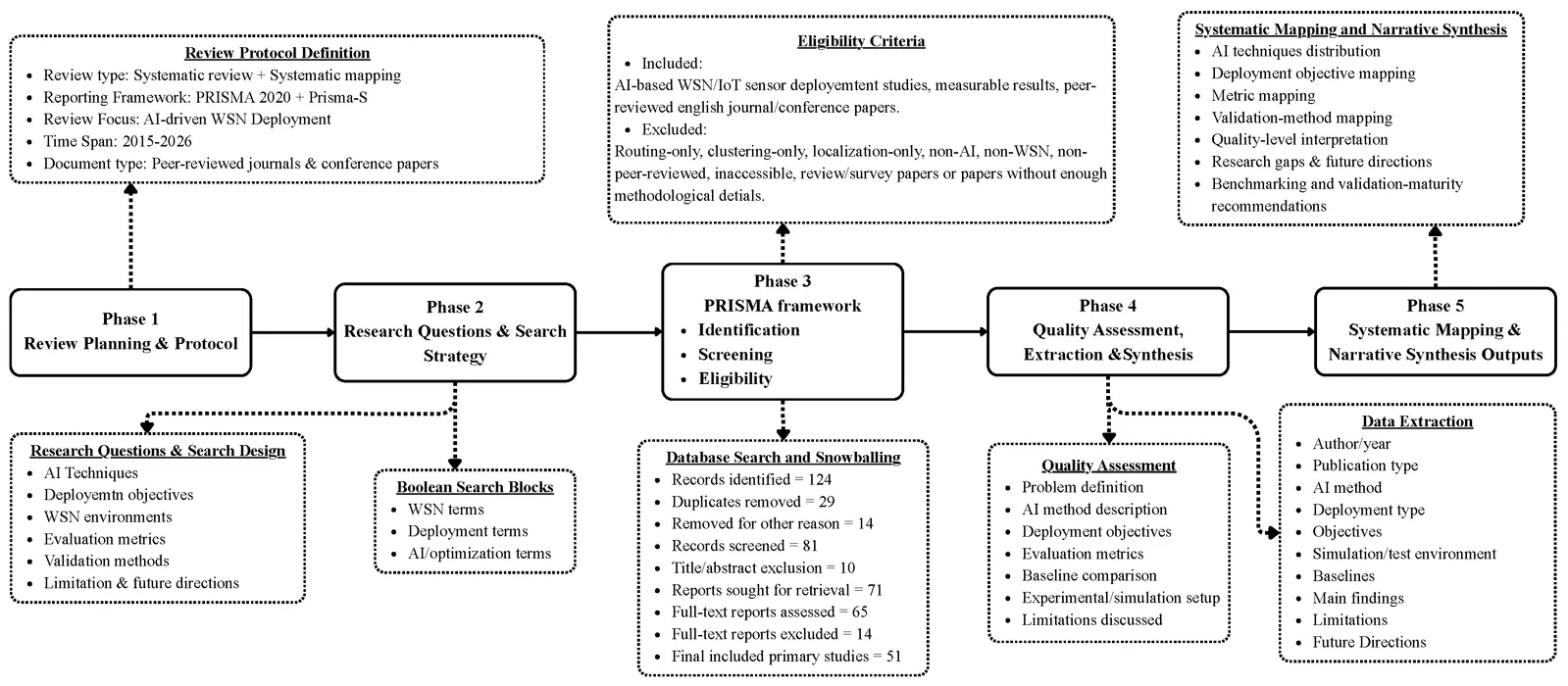
Methodological workflow for the PRISMA-guided systematic review and systematic mapping of AI-driven WSN deployment studies. The workflow illustrates review protocol development, research-question and search-strategy design, database searching and snowballing, PRISMA-based study selection, eligibility assessment, reporting-completeness appraisal, data extraction, and final narrative synthesis outputs.

**Figure 2:**
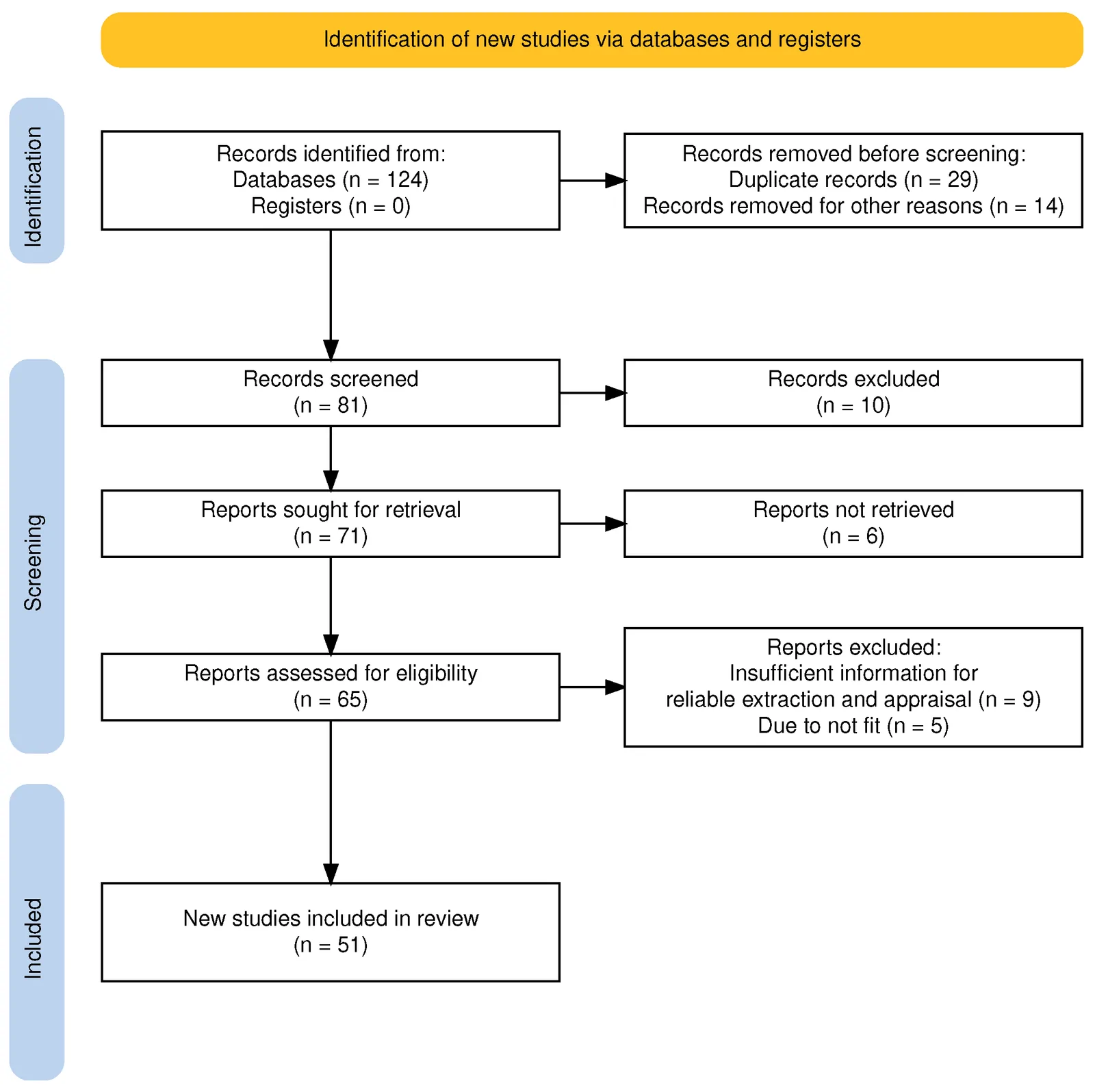
PRISMA flow diagram showing the identification, screening, eligibility assessment, and final inclusion of studies in the systematic review.

**Table 1:** Scope and methodological emphasis of selected prior reviews relative to the present review.

Study	Main Focus	PRISMA Flow	AI-Driven Deployment	Validation Taxonomy	Reporting-Completeness Appraisal	Benchmarking	Edge Feasibility
[1]	General WSN architecture and design constraints	Not stated	Not stated	Not stated	Not stated	Not stated	Discussed
[13]	Energy-efficient WSN coverage	Not stated	Not stated	Not stated	Not stated	Not stated	Not stated
[4]	Coverage and connectivity in WSNs	Not stated	Not stated	Not stated	Not stated	Not stated	Not stated
[5]	Energy-efficient coverage protocols	Not stated	Not stated	Not stated	Not stated	Not stated	Not stated
[9]	Coverage protocols and future directions	Not stated	Discussed	Not stated	Not stated	Discussed	Not stated
[14]	Smart-city deployment and management	Not stated	Discussed	Discussed	Not stated	Discussed	Discussed
[6]	Machine learning in WSNs	Not stated	Discussed	Not stated	Not stated	Not stated	Discussed
[10]	Smart-city deployment optimization	Discussed	Discussed	Not stated	Discussed	Discussed	Not stated
[7]	AI for coverage, deployment, and localization	Not stated	Primary focus	Discussed	Not stated	Discussed	Discussed
[8]	AI-based WSN deployment and objective modeling	Not stated	Primary focus	Discussed	Not stated	Discussed	Discussed
[16]	Heuristics and metaheuristics for WSNs	Not stated	Discussed	Not stated	Not stated	Discussed	Not stated
[11]	Metaheuristic methods in WSNs	Not stated	Discussed	Not stated	Not stated	Discussed	Discussed
[17]	RL and DRL in wireless/mobile networking	Not stated	Discussed	Not stated	Not stated	Not stated	Discussed
[12]	Deep learning for WSN deployment	Not stated	Discussed	Discussed	Not stated	Discussed	Primary focus
**This review**	**AI-driven WSN deployment across methods, objectives, environments, validation, reporting completeness, and research gaps**	**Primary focus**	**Primary focus**	**Primary focus**	**Primary focus**	**Primary focus**	**Discussed**

*Note:* The comparison describes the explicitly stated scope and analytical emphasis of each review and is not intended as a quality ranking. “Primary focus” indicates that the dimension is explicitly incorporated into the review scope, taxonomy, methodology, or structured synthesis. “Discussed” indicates that the topic is considered but not systematically coded or assessed as a review dimension. “Not a stated review dimension” indicates that the dimension is not explicitly presented as part of the review’s analytical structure.

**Table 2:** Review protocol and operationalization of the systematic mapping process. The table summarizes the methodological scope of the review, including the reporting framework, search period, evidence sources, eligibility boundaries, screening procedure, reporting-completeness appraisal approach, data-extraction strategy, and synthesis method.

Protocol Element	Operationalization in This Review
Review type	Systematic review with systematic mapping
Reporting framework	PRISMA 2020 and PRISMA-S
Review focus	AI-driven wireless sensor deployment in WSNs and closely related IoT sensor-network contexts
Time span	2015 to 2026
Evidence sources	IEEE Xplore, ACM Digital Library, ScienceDirect, SpringerLink, MDPI, Nature, Taylor & Francis, and Wiley Online Library; Google Scholar for snowballing only
Language	English
Document types	Peer-reviewed journal articles and full conference papers
Screening stages	Identification, duplicate removal, title/abstract screening, full-text eligibility, final inclusion
reporting-completeness appraisal	Seven-item binary checklist
Data handling	Standardized data-extraction form
Synthesis	Descriptive statistics, classification tables, and thematic narrative synthesis

**Table 3:** Research questions guiding the systematic review and mapping analysis. The questions define the analytical scope of the study by covering AI techniques, deployment objectives, WSN environments and application domains, evaluation metrics, validation practices, and the main limitations and future directions reported in the literature.

Code	Research Question
RQ1	What AI techniques are used for wireless sensor deployment optimization?
RQ2	What deployment objectives are addressed in the selected studies?
RQ3	What WSN environments and application domains are considered?
RQ4	What performance metrics are used to evaluate AI-driven deployment methods?
RQ5	What validation methods, simulators, datasets, and baselines are used in the reviewed studies?
RQ6	What major limitations, challenges, and future research directions emerge from the current literature?

**Table 4:** Database-specific search expressions, search fields, and retrieval settings used for study identification.

Database	Representative Search Syntax or Field Logic
Scopus	TITLE-ABS-KEY((“wireless sensor network” OR WSN OR “sensor network”) AND (deployment OR placement OR “node placement” OR “sensor placement” OR redeployment OR “coverage optimization”) AND (“artificial intelligence” OR “machine learning” OR “deep learning” OR “reinforcement learning” OR “genetic algorithm” OR “particle swarm optimization” OR metaheuristic))
Web of Science	TS=((“wireless sensor network” OR WSN OR “sensor network”) AND (deployment OR placement OR “node placement” OR “sensor placement” OR redeployment OR “coverage optimization”) AND (“artificial intelligence” OR “machine learning” OR “deep learning” OR “reinforcement learning” OR “genetic algorithm” OR “particle swarm optimization” OR metaheuristic))
IEEE Xplore, ACM Digital Library, ScienceDirect, SpringerLink, MDPI, Nature, Taylor & Francis and Wiley Online Library	Search in All Metadata using the same three concept blocks and Boolean structure
Google Scholar	Used only for backward and forward snowballing of included studies and key review papers

**Table 5:** Methodological-reporting completeness checklist applied to empirical studies.

Code	Quality Question	Score
QA1	Is the deployment problem clearly defined?	0/1
QA2	Is the AI method clearly described?	0/1
QA3	Are the deployment objectives clearly stated?	0/1
QA4	Are evaluation metrics reported?	0/1
QA5	Are baseline methods used for comparison?	0/1
QA6	Is the simulation or experimental setup clearly explained?	0/1
QA7	Are limitations discussed?	0/1

**Table 6:** Methodological-reporting completeness scores for included empirical studies.

Study	QA1	QA2	QA3	QA4	QA5	QA6	QA7	Total	Level
[31]	1	1	1	1	1	1	0	6	High
[32]	1	1	1	1	1	1	0	6	High
[33]	1	1	1	1	0	1	0	5	Medium
[34]	1	1	1	1	1	1	0	6	High
[35]	1	1	1	1	1	0	0	5	Medium
[36]	1	1	1	1	0	1	0	5	Medium
[37]	1	0	1	1	1	1	0	5	Medium
[38]	1	0	1	1	0	1	1	5	Medium
[39]	1	1	1	1	1	1	0	6	High
[40]	1	1	1	1	0	1	0	5	Medium
[41]	1	1	1	1	1	1	1	7	High
[42]	1	1	1	1	1	1	0	6	High
[43]	1	0	1	0	0	0	1	3	Low
[44]	1	1	1	1	1	1	1	7	High
[45]	1	1	1	1	1	1	0	6	High
[46]	1	1	1	1	1	1	1	7	High
[47]	1	1	1	1	0	1	1	6	High
[48]	1	1	1	1	1	1	1	7	High
[49]	1	1	1	1	1	1	0	6	High
[50]	1	1	1	1	1	1	1	7	High
[51]	1	1	1	1	1	1	0	6	High
[52]	1	1	1	1	0	1	1	6	High
[53]	1	1	1	1	1	0	0	5	Medium
[54]	1	1	1	1	1	1	0	6	High
[55]	1	1	1	1	1	1	1	7	High
[56]	1	1	1	1	1	1	1	7	High
[57]	1	1	1	1	1	1	0	6	High
[58]	1	1	1	1	1	1	0	6	High
[59]	1	1	1	1	1	1	1	7	High
[60]	1	1	1	1	1	1	0	6	High
[61]	1	1	1	1	1	1	0	6	High
[62]	1	1	1	1	1	1	0	6	High
[63]	1	1	1	1	1	1	0	6	High
[64]	1	1	1	1	1	1	0	6	High
[65]	1	1	1	1	1	1	0	6	High
[66]	1	1	1	1	1	1	0	6	High
[67]	1	1	1	1	1	1	0	6	High
[68]	1	0	1	0	0	0	1	3	Low
[69]	1	1	1	1	1	1	0	6	High
[70]	1	1	1	1	1	1	0	6	High
[71]	1	1	1	1	1	1	0	6	High
[72]	1	1	1	1	1	1	1	7	High
[73]	1	1	1	1	0	1	0	5	Medium
[74]	1	1	1	1	0	1	0	5	Medium
[75]	1	1	1	1	0	1	1	6	High
[76]	1	1	1	1	1	1	0	6	High
[77]	1	1	1	1	1	0	0	5	Medium
[78]	1	1	1	1	0	1	0	5	Medium
[79]	1	1	1	1	0	1	0	5	Medium
[80]	1	1	1	1	1	1	0	6	High
[81]	1	1	1	1	0	1	0	5	Medium

*Note:* QA1–QA7 represent the seven methodological-reporting completeness criteria. Scores of 6–7, 5, and 0–4 correspond to High, Medium, and Low reporting completeness, respectively.

**Table 7:** Hierarchical, multi-label distribution of AI techniques and methodological descriptors across the included studies.

Technique Family/Descriptor	Studies	References
*Optimization-based method families*		
Genetic and evolutionary algorithms	6	[36,53,56,64,72,80]
Swarm-based and nature-inspired metaheuristics	8	[39,44,48,55,57,66,67,69]
Bayesian optimization	1	[32]
*Learning-based method families*		
Machine learning	12	[31,33,35,37,40,51,52,54,73,75,79,81]
Deep learning	13	[37,45,46,47,54,57,58,59,62,65,70,74,78]
Reinforcement learning	10	[34,45,58,59,60,61,71,76,77,79]
*Cross-cutting components and descriptors*		
Fuzzy logic	1	[48]
Graph-based learning/similarity	2	[42,58]
Hybrid methods	8	[41,48,50,58,72,76,77,79]
*Other or contextual approaches*		
Other deployment and contextual studies	3	[38,43,68]

*Note:* Categories are non-mutually exclusive. Studies combining multiple methodological components may appear in more than one category. Hybrid methods represent a cross-cutting descriptor rather than an independent algorithm family. Counts denote distinct studies within each row and should not be summed across rows. The “Other” category is not a residual metaheuristic family.

**Table 8:** Frequency of deployment objectives addressed across the included studies (non-mutually exclusive classification).

Deployment Objective	Number of Studies	Studies
Coverage maximization	31	[31,32,34,36,38,39,41,42,44,48,49,55,56,57,58,61,63,64,65,66,67,68,69,73,75,76,77,78,79,80,81]
Connectivity preservation/availability	12	[31,34,37,43,48,56,64,66,68,75,76,80]
Energy efficiency	19	[31,32,33,34,48,56,58,61,64,65,66,67,68,69,73,76,77,78,81]
Network lifetime	13	[31,32,34,48,56,61,64,65,67,68,69,73,77]
Cost reduction/node-count reduction/deployment-effort reduction	8	[38,45,52,60,64,70,78,80]
Fault tolerance/robustness/dependability	12	[38,42,44,48,49,52,55,56,58,69,75,80]
Latency reduction/detection-time reduction	5	[37,45,60,65,67]
Load balancing/energy balancing	3	[48,61,64]
Localization/positioning accuracy	4	[37,49,50,65]
Redundancy reduction/overlap reduction/node contribution	9	[33,42,46,51,55,58,62,79,80]
Application-specific sensing, reconstruction, classification, or damage-detection accuracy	9	[52,53,54,59,60,62,70,71,72]
Topology observability/topology identification	1	[74]

*Note:* A study may appear in multiple objective categories but is counted only once within each category. The 31 studies associated with coverage maximization include studies addressing coverage in any coded role, not necessarily as their primary objective. Primary and secondary objective priorities were not separately quantified. Therefore, the category totals should not be summed or interpreted as a ranking of primary optimization objectives.

**Table 9:** Distribution of deployment environments and application domains represented in the included studies.

Deployment Environment/Application Domain	Number of Studies	Studies
2D WSN	24	[31,32,33,34,36,39,42,44,48,49,55,56,58,61,63,64,65,66,67,73,76,77,78,81]
3D WSN/spatial deployment	6	[41,45,46,51,68,80]
Static WSN/planned initial deployment	19	[33,36,39,41,42,44,46,48,49,51,55,56,63,64,66,67,68,76,80]
Mobile, dynamic, or adaptive deployment	12	[31,34,50,58,61,65,69,73,77,78,79,81]
UAV-assisted, autonomous, or robot-assisted sensing	1	[57]
Underwater/aquatic monitoring context	2	[57,69]
Industrial IoT/smart production	3	[37,43,79]
Smart city/transportation/urban infrastructure	5	[35,45,52,63,75]
Environmental monitoring/on-demand deployment	5	[38,52,57,60,69]
Smart home/healthcare/assisted living/wearable sensing	7	[40,47,53,54,59,62,63]
Structural health monitoring/modal testing/pipeline systems	4	[46,51,70,72]
Water infrastructure and water-quality monitoring	2	[52,60]
Power-system and active distribution-network monitoring	1	[74]
Spatiotemporal process modeling/distributed parameter systems	1	[71]
Smart spaces/IoT indoor environments	1	[80]

**Table 10:** Validation methods reported across the included studies.

Validation Method	Number of Studies	Studies
Simulation or custom computational evaluation	33	[31,32,33,34,36,38,39,41,42,44,45,48,49,50,55,56,57,58,61,63,64,65,66,67,69,73,75,76,77,78,79,80,81]
Benchmark-based or dataset-based evaluation	17	[35,37,40,43,47,51,52,53,54,55,59,60,62,70,73,74,75]
Real testbed or controlled physical experiment	5	[47,53,54,59,71]
Field deployment	1	[47]
Digital twin, finite-element, hydraulic, structural, or numerical-model validation	9	[46,51,52,60,68,70,71,72,74]
Mathematical analysis or exact-optimization comparison	6	[48,49,60,71,76,79]

*Note:* Validation categories are non-mutually exclusive. A study may appear in multiple categories when more than one validation method was used. Counts represent distinct studies within each category and should not be summed across rows.

**Table 11:** Cross-method comparative synthesis of AI-driven WSN deployment studies.

AI Method Family	Representative Studies	Dominant Deployment Use	Common Evaluation Metrics	Main Validation Pattern	Comparative Interpretation
Genetic and evolutionary algorithms	[36,53,60,64,80]	Combinatorial placement, many-objective optimization, sensor selection, and 3D self-deployment	Coverage, connectivity, cost, load balancing, detection time, convergence	Simulation, datasets, and controlled experiments	Effective for discrete and combinatorial placement; scalability depends on encoding and search-space size
Swarm intelligence and nature-inspired metaheuristics	[39,44,48,63,66,67,69]	Static and target coverage, energy-aware placement, underwater deployment, and repositioning	Coverage, connectivity, energy, lifetime, movement distance, throughput, packet delivery, convergence	Predominantly simulation-based	Strongest evidence for classical coverage optimization, but practical claims remain limited by simulation dependence and inconsistent assumptions
Fuzzy and rule-based components	[48]	Uncertainty-aware, energy-aware, and load-balancing support	Coverage, connectivity, energy, lifetime	Simulation and analytical evaluation	Most useful as a support mechanism combined with optimization methods
Reinforcement learning and deep reinforcement learning	[34,45,65,71,76,77,79]	Adaptive redeployment, mobile WSNs, sequential placement, IIoT, spatiotemporal modeling, and dynamic coverage	Coverage, connectivity, reward, energy, movement cost, error, latency, lifetime	Mostly simulation or dataset/model-based validation	Best suited to adaptive and sequential deployment; performance depends strongly on reward design, state/action representation, and training realism
Machine learning	[33,35,37,40,52,54,73,81]	Prediction-supported, localization-aware, human-sensing, drainage, and dynamic placement	Prediction and placement accuracy, localization error, coverage, packet delivery, energy, redundancy	Dataset, simulation, and application-specific evaluation	Strong for data-driven and task-specific placement, but not directly comparable with classical coverage optimization
Deep learning	[37,46,58,62,65,70,75,78]	High-dimensional, topology-aware, reconstruction, dependability, and application-specific placement	Recognition, reconstruction and prediction error, coverage, energy, topology accuracy, latency, throughput	Dataset, simulation, numerical-model, and application-specific evaluation	Strong for complex data-driven placement; training cost and generalization remain key limitations
Hybrid AI and optimization methods	[41,48,50,58,60,65,67,70,72,76,77,80]	Multi-objective, topology-aware, localization-aware, 3D, structural, and adaptive deployment	Coverage, connectivity, energy, cost, lifetime, localization and modeling error, stability	Simulation, benchmarks, datasets, and case studies	Promising for realistic multi-objective deployment, but stronger ablation and benchmarking are required
Bayesian and surrogate-assisted optimization	[32,70]	Black-box optimization and expensive placement evaluation	Objective value, sensor count, damage accuracy, coverage, utility	Simulation and structural-model case studies	Useful when candidate evaluation is expensive, although the evidence base remains limited
Graph-based, topology-aware, centrality-based, and explainable-AI methods	[42,58,72,74,76]	Topology-aware deployment, redundancy analysis, observability, search reduction, and critical-node identification	Coverage, robustness, topology similarity, observability, cost, connectivity, energy	Simulation, model-based, and application-specific validation	Important for topology and interpretability, but evidence remains emerging and methodologically diverse
Conceptual, and framework-oriented studies	[43,68]	Deployment guidance, 3D synthesis, validation gaps, and future directions	Qualitative and methodological criteria	Conceptual analysis	Useful for interpretation and gap identification, but not direct evidence of algorithmic effectiveness

**Table 12:** Proposed benchmarking requirements for AI-driven WSN deployment studies.

Benchmarking Dimension	Required Benchmark Elements	Purpose
Deployment scenarios	2D open-field; obstacle-aware 2D; 3D; indoor smart home; outdoor monitoring; industrial IoT; smart-city roadside; underwater/aquatic; UAV-assisted sensing	Tests performance across simple and realistic environments
Node assumptions	Homogeneous and heterogeneous static nodes; mobile sensors or sinks; UAV-assisted, energy-constrained, and failure-prone nodes	Tests generalization beyond ideal static networks
Sensing models	Binary, probabilistic, directional, obstruction-aware, and range-degraded sensing	Avoids reliance on ideal circular sensing
Communication models	Fixed range; RSSI/path-loss; interference-aware; packet-loss-aware; multi-hop communication	Captures realistic data-delivery constraints
Evaluation metrics	Coverage; k-coverage; k-connectivity; residual energy; lifetime; localization error; packet delivery; latency; computational and deployment cost; robustness; fault tolerance	Supports multi-objective comparison
Baseline algorithms	Random and grid deployment; virtual force; GA; PSO; ACO; fuzzy logic; RL; ML; application-specific existing layouts	Prevents claims based on weak baselines
Reproducibility artifacts	Source code; seeds; parameter tables; datasets; scenario maps; node coordinates; configuration files; baseline implementations; hardware details; train-test splits; evaluation scripts	Supports replication and fair comparison

**Table 13:** Illustrative deployment-scenario templates proposed for future benchmarking.

Scenario Code	Scenario Type	Minimum Configuration
**S1**	2D open-area WSN	Rectangular area, homogeneous nodes, binary sensing, static sink
**S2**	2D obstacle-aware WSN	Obstacles, blocked sensing regions, path-loss communication
**S3**	3D WSN	3D deployment volume, height constraints, 3D sensing
**S4**	Directional sensor network	Directional field of view, orientation constraints, occlusion
**S5**	Indoor smart-home WSN	Room topology, walls, activity zones, privacy-sensitive sensors
**S6**	Industrial IoT	Interference, metallic obstacles, latency constraints, predictive-maintenance objective
**S7**	Smart-city/roadside sensing	Road geometry, moving targets, occlusion, multi-sensor coverage
**S8**	Underwater/aquatic sensing	Communication degradation, sparse deployment, drift or current effects
**S9**	UAV-assisted WSN	Mobile aerial nodes, limited flight time, dynamic coverage, redeployment cost
**S10**	Failure-aware WSN	Node failure, battery depletion, coverage repair, redundancy requirements

**Table 14:** Conceptual lifecycle-oriented taxonomy derived from the reviewed AI-driven WSN deployment literature.

Lifecycle Stage	Main Purpose	Typical Methods or Evidence	Current Weakness
Application and environment characterization	Define task, environment, node assumptions, and operational constraints	Environment modeling, sensing assumptions, candidate locations, application analysis	Simplified 2D settings often omit obstacles, terrain, RF irregularity, mobility, hardware, and installation constraints
Objective and constraint formulation	Define measurable deployment objectives and constraints	Coverage, connectivity, energy, lifetime, localization, latency, cost, robustness, privacy, edge and task-level requirements	Coverage and energy dominate; robustness, security, privacy, uncertainty, latency, and lifecycle requirements receive less attention
AI-method selection	Match the AI family to the deployment problem	Swarm and evolutionary methods, RL, ML, DL, Bayesian optimization, graph methods, XAI, and hybrids	Selection is sometimes driven by algorithmic novelty rather than explicit suitability to the deployment context
Deployment optimization	Generate placement, redeployment, or sensor-selection decisions	Static and mobile placement, target coverage, 3D and directional deployment, topology-aware allocation	Reproducible assumptions, parameters, maps, code, and implementation artifacts are often limited
Validation Design and Evidence Characterization	Characterize the type, realism, independence, and operational relevance of validation evidence	Simulation, benchmark datasets, numerical models, digital twins, testbeds, field deployments, long-term experiments	Simulation dominates, while physical and long-term validation remain scarce
Benchmarking and reproducibility	Enable fair comparison and replication	Shared scenarios, metrics, baselines, datasets, source code, seeds, sensing and communication models	Lack of standardized benchmarks limits reliable cross-method comparison
Lifecycle adaptation	Maintain sensing performance under degradation and changing requirements	Self-healing coverage, recalibration, adaptive scheduling, redeployment, online learning, maintenance-aware optimization	Most studies treat deployment as an initial placement problem rather than a lifecycle process

The taxonomy summarizes recurring evidence patterns and methodological gaps. It is not a validated checklist, scoring rubric, or study-ranking instrument.

## Data Availability

No new experimental data were generated in this systematic review. The analysis is based on information extracted and coded from 51 published primary studies, which are identified in the reference list. The study-level methodological-reporting completeness scores and the classification results reported in this review are presented in Table 6, Table 7, Table 8, Table 9 and Table 10.
